# Dual‐Targeting Biomimetic Nanozymes Loaded Microneedle Patch Promotes Scarless Wound Healing Through Anti‐Inflammatory and Anti‐Fibrotic Effects

**DOI:** 10.1002/advs.77192

**Published:** 2026-08-17

**Authors:** Hongyi Zhang, Jinwei Li, Shan Hua, Shengming Wu, Chenlong He, Yuan Zhu, Ming Yin, Han Zhou, Huawei Liu, Chika Hasegawa, Jun Lyu, Keyue Xiao, Hua Jiang, Yilong Wang, Yuxin Qian

**Affiliations:** ^1^ Department of Plastic Surgery Ear Reconstruction Institute Shanghai East Hospital School of Medicine Tongji University Shanghai People's Republic of China; ^2^ Department of Pediatric Neurosurgery Xinhua Hospital Affiliated to Shanghai Jiao Tong University School of Medicine Shanghai People's Republic of China; ^3^ Department of Plastic Surgery Ear Reconstruction Institute State Key Laboratory of Cardiology and Medical Innovation Center Shanghai East Hospital The Institute for Biomedical Engineering & Nano Science School of Medicine Tongji University Shanghai People's Republic of China; ^4^ Massachusetts General Hospital Harvard University Boston Massachusetts USA

**Keywords:** anti‐fibrosis, fibroblast activation protein, inflammation, nanozymes, skin wound healing

## Abstract

During skin wound healing, complex interactions among multiple cell types and dynamic phase evolution make it difficult for biomaterial scaffolds to balance late‐healing and over‐healing, often resulting in delayed wound healing, persistent inflammatory responses, and hypertrophic scars due to spatiotemporal mismatch. Precise regulation of fibroblast‐to‐myofibroblast transformation and macrophage polarization is therefore essential yet challenging. Fibroblast activation protein (FAP) has been identified as a key bio‐cue in pulmonary and hepatic fibrosis, but whether FAP‐targeting peptide (FTP) within a biomimetic system can address this dilemma remains unclear. Here, a methacrylated hyaluronic acid hydrogel microneedle patch (CAF@MN) integrating a dual‐targeting biomimetic nanozyme system (Cu‐CeO_2_@ABs‐FTP) modified with apoptotic bodies (ABs) membranes and FTP is developed. Cu‐CeO_2_ nanocomposites activate fibroblast functions and exhibit anti‐inflammatory, antibacterial, antioxidant, and pro‐angiogenic activities. The ABs membrane enables macrophage targeting and M2 polarization, while FTP specifically modulates myofibroblast activation. The microneedle architecture enhances deep local delivery and nanozyme utilization. In mouse and rabbit full‐phase wound models, the system achieves accelerated wound healing, reduced inflammation, and attenuated hypertrophic scars. Single‐cell RNA sequencing further elucidates the dual‐targeted regulatory mechanisms underlying anti‐inflammation and scar suppression.

## Graphical abstract

This study developed a dual‐targeting biomimetic nanozyme hybrid system (Cu‐CeO_2_@ABs‐FTP) and integrated it with HAMA hydrogel to fabricate a microneedle patch (CAF@MN). The patch promotes fibroblast‐mediated wound repair, targets macrophages to induce M2 polarization, and targets myofibroblasts to exert anti‐fibrotic effects, thereby enabling rapid, anti‐inflammatory, and scarless wound healing.

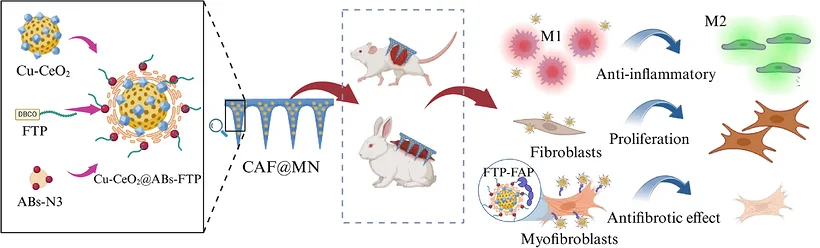

**Abbreviations**
•OH: hydroxyl radical
ABs: apoptotic bodies
ALT: alanine aminotransferase
BF: bright field
CAT: catalase
CK: creatine kinase
CM: conditioned medium
DBCO: dibenzocyclooctyne
DEGs: differentially expressed genes
DLS: dynamic light scattering
E. coli: *Escherichia coli*
ECM: extracellular matrix
EDS: energy dispersive x‐ray spectroscopy
FAP: fibroblast activation protein
FTP: fibroblast activation protein‐targeting peptide
GLU: blood glucose
GO‐BP: gene ontology—biological process
GSEA: gene set enrichment analysis
GSH: reduced glutathione
H_2_O_2_
: hydrogen peroxide
HAADF: high‐angle annular dark‐field
HAMA: methacrylated hyaluronic acid
HDFs: human dermal fibroblasts
HSFs: human skin fibroblasts
KEGG: Kyoto encyclopedia of genes and genomes
LPS: lipopolysaccharide
MN: microneedle
MRSA: methicillin‐resistant *Staphylococcus aureus*
N_3_
: azide groups
NO: nitric oxide
O_2_•^−^
: superoxide anion
PS: phosphatidylserine
PVA: poly (vinyl alcohol)
ROS: reactive oxygen species
RT‐qPCR: real‐time quantitative polymerase chain reaction
scRNA‐seq: single‐cell rna sequencing
SDS‐PAGE: sodium dodecyl sulfate‐polyacrylamide gel electrophoresis
SEI: scar elevation index
SEM: scanning electron microscopy
STEM: scanning transmission electron microscopy
STS: staurosporine
T‐AOC: total antioxidant capacity
TEM: transmission electron microscopy
TG: triglycerides
T‐SOD: total superoxide dismutase
UMAP: uniform manifold approximation and projection
UREA: urea

## 1 Introduction

After skin injury, dysfunction of fibroblasts, myofibroblasts, and macrophages, and uncontrolled cellular interactions lead to delayed wound healing, persistent inflammatory responses, and hypertrophic scars, thereby severely compromising the quality of skin repair [1, 2, 3, 4]. Therefore, in situ targeting of dominated cells’ status and precise regulation of their behaviors during the full‐phase covered skin wound healing are of great significance. Currently, this field is primarily constrained by three key aspects: first, insufficient regulation across the entire wound healing process. For instance, there is often an overemphasis on promoting early fibroblast‐mediated granulation tissue formation to fill the wound, while the role of myofibroblasts in wound closure during later stages is neglected [5, 6]. Second, immune cell‐mediated inflammatory complications, as the wound inflammatory microenvironment readily induces macrophage polarization toward the M1 phenotype, thereby impairing wound healing [7]. Third, the absence of precise identification of specific cell types. Failure to target excessively activated myofibroblasts during the middle and late stages of wound healing can lead to hypertrophic scarring [8, 9]. Collectively, these findings indicate that, based on comprehensive regulation of the entire wound healing phase, the precise identification and modulation of multiple functional cell types remain critical challenges.

Some recent studies have achieved a dynamic balance between “insufficient healing” and “excessive fibrosis” by designing multiple nanomaterials to simultaneously regulate fibroblasts and myofibroblasts, thereby suppressing hypertrophic scars from different aspects [9, 10, 11]. Nanozyme, as a powerful tool for reactive oxygen species (ROS) production or inhibition, has good potential for controlling persistent inflammatory responses and oxidative stress, which is pivotal for scarless wound healing. However, the existing biomaterials systems seldom possess the capacity for targeted recognition of inflammatory regions and myofibroblasts activation. Notably, fibroblasts can act as both “friend” and “foe,” and the inability to selectively identify and suppress hyperactive fibroblasts is one of the major causes of hypertrophic scars [5]. Thus, targeted identification and precised regulation of fibroblasts to myofibroblasts transformation and over‐activation, and macrophages are urgently desired.

Theoretically, the Gly‐Pro sequence within the fibroblast activation protein‐targeting peptide (FTP) can specifically recognize and be cleaved by fibroblast activation protein (FAP), a protein highly expressed on the surface of excessively activated myofibroblasts [12, 13, 14, 15], which has been investigated in pulmonary and hepatic fibrosis treatment [16, 17, 18]. However, it is yet to be known whether FTP involved biomimetic system could realize duly and precise regulation of fibroblasts behaviors for achieving scarless skin wound healing. Additionally, membranes derived from apoptotic bodies (ABs) of Jurkat T cells are considered to possess strong targeting capability toward inflammatory regions. Their mimic apoptotic clearance signals are rapidly recognized and engulfed by macrophages, thereby promoting macrophage M2 polarization and suppressing the progression of wound inflammation [19, 20]. With these promising cell targeting cues, other moieties are also necessary for constructing a multifunctional biomimetic system. Such as, copper (Cu) has been proven to exhibit favorable antibacterial properties [21], while hydrogel microneedle (MN) patches can enhance local drug utilization efficiency [22]. To explore the underlying mechanism, single‐cell RNA sequencing (scRNA‐seq) is beneficial for analyzing key cell populations and their functional state changes in wound tissues following biomaterial treatment.

In this study, to face key challenges in wound healing—delayed wound healing, persistent inflammatory responses, and hypertrophic scars, a system composed of well‐organized multiple moieties, including CeO_2_ nanozymes, Cu, FTP, and ABs membrane, was developed to achieve a comprehensive effect of “promoting repair, controlling inflammation, and suppressing hypertrophic scars” in the skin wound tissue regeneration. In detail, first, Cu‐CeO_2_ nanoparticles with antioxidant and antibacterial properties were synthesized. ABs were extracted from Jurkat T cells and modified with azide groups (N_3_) to obtain ABs‐N_3_. ABs‐N_3_ were first coated onto the surface of Cu‐CeO_2_ nanoparticles through liposome extrusion to obtain Cu‐CeO_2_@ABs‐N_3_. Subsequently, DBCO‐modified FTP was conjugated to the externally accessible N_3_ groups on the nanoparticle surface via copper‐free click chemistry, thereby constructing the Cu‐CeO_2_@ABs‐FTP biomimetic nanozyme hybrid system. Subsequently, the nanoparticles were mixed with methacrylated hyaluronic acid (HAMA) hydrogel and fabricated into microneedle patches containing Cu‐CeO_2_@ABs‐FTP using a mold‐based method, termed CAF@MN (Scheme 1A). In vitro and in vivo experiments demonstrated that the nanoparticles exhibited favorable antibacterial, antioxidant, anti‐inflammatory, and pro‐angiogenic properties, as well as targeting capability toward macrophages in inflammatory regions [23, 24] (Scheme 1B). Meanwhile, the targeting ability of the nanoparticles toward myofibroblasts was also validated. During the early and middle stages of wound healing, the nanoparticles acted on and activated fibroblasts, promoting their proliferation and type III collagen secretion, thereby accelerating wound healing [25] (Scheme 1C). In the late stage of wound healing, benefiting from the FTP‐mediated specific recognition of FAP, the nanoparticles were able to selectively target and inhibit excessively activated myofibroblasts, suppressing their excessive contraction and type I collagen secretion, thus effectively alleviating hypertrophic scar (Scheme 1D). Finally, scRNA‐seq was used to validate the above findings at the single‐cell level.

**SCHEME 1:**
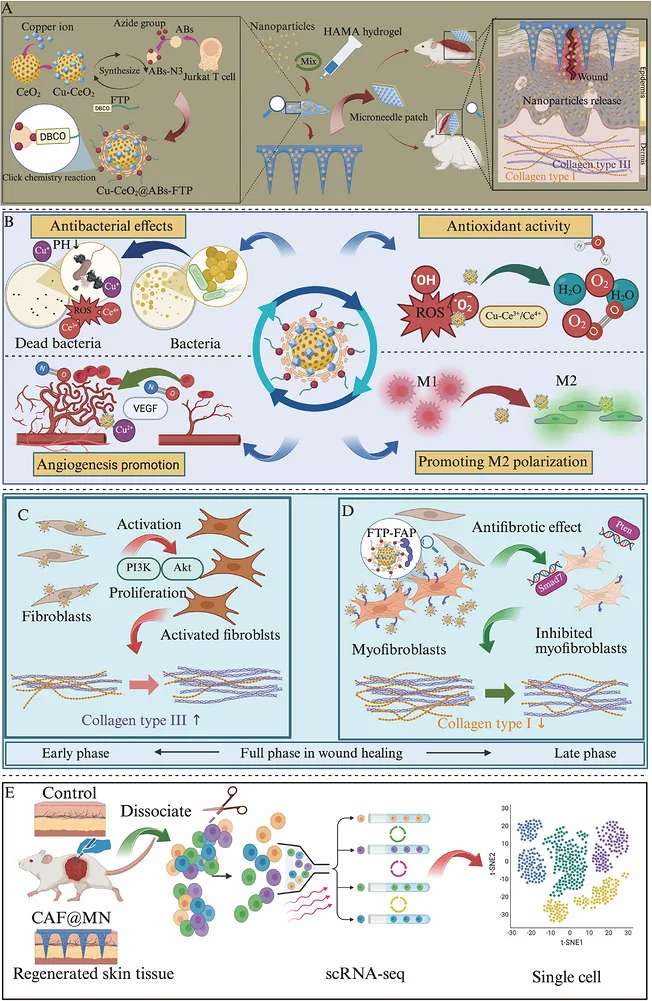
(A) Schematic illustration of the preparation of the Cu‐CeO_2_@ABs‐FTP dual‐targeting biomimetic nanozyme hybrid system, the fabrication of HAMA hydrogel microneedle patches containing the nanoparticles (CAF@MN), azide‐modified apoptotic bodies (ABs‐N_3_), DBCO‐modified fibroblast activation protein‐targeting peptide (FTP), the click chemistry reaction between dibenzocyclooctyne (DBCO) and N_3_, and the application of CAF@MN in mouse and rabbit wound models; (B) Throughout the entire wound healing process, the nanoparticles exert antibacterial, antioxidant, anti‐inflammatory, and pro‐angiogenic effects. In particular, via the ABs membrane, the nanoparticles target inflammatory regions and promote macrophage polarization toward the M2 phenotype; (C) During the early stage of wound healing, the nanoparticles activate fibroblast functions by regulating the PI3K‐AKT pathway and increase type III collagen deposition to support regenerative repair; (D) During the late stage of wound healing, the nanoparticles specifically recognize fibroblast activation protein (FAP) expressed on the surface of myofibroblasts via the FTP, and suppress excessive myofibroblast activation by modulating key genes such as Smad7, thereby reducing type I collagen deposition and inhibiting scar formation via antifibrotic effect; (E) scRNA‐seq analysis of regenerated mouse wound tissues identifies key cell subsets and molecular mechanisms, validating that the nanoparticles promote high‐quality wound healing at single‐cell resolution.

The results showed that after CAF@MN treatment, fibroblast functions were activated, myofibroblasts were suppressed, and macrophages exhibited a marked polarization toward the M2 phenotype in the experimental group. In addition, pseudotime analysis was performed to investigate the dynamic temporal evolution of these cells and genes, and mechanistic analysis suggested that CAF@MN‐mediated fibroblast activation may be driven by PI3K‐AKT [26, 27, 28], whereas myofibroblast inhibition was associated with genes such as Smad7 and Pten (Scheme 1E).

## 2 Results and Discussion

### 2.1 Synthesis and Characterization of Cu‐CeO_2_@ABs‐FTP Nanoparticles

First, CeO_2_ nanoparticles (Figures S1 and S2) and Cu‐CeO_2_ nanoparticles (Figure 1A) were synthesized in this study. Figure S3 illustrates the preparation procedure of the nanoparticles. Briefly, SiO_2_ cores were first synthesized via the Stöber method, followed by the formation of cerium oxide on the silica surface using a cerium acetylacetonate‐based hydrothermal approach. During the reaction, the SiO_2_ nanoparticle cores were gradually dissolved, thereby avoiding harsh post‐treatment steps such as strong alkali etching. Subsequently, ultrasmall Cu nanoparticles were prepared through the classical vitamin C‐mediated reduction of copper chloride [21]. Finally, the Cu nanoparticles were combined with CeO_2_ nanoparticles via electrostatic adsorption to obtain Cu‐CeO_2_ nanoparticles.

**FIGURE 1:**
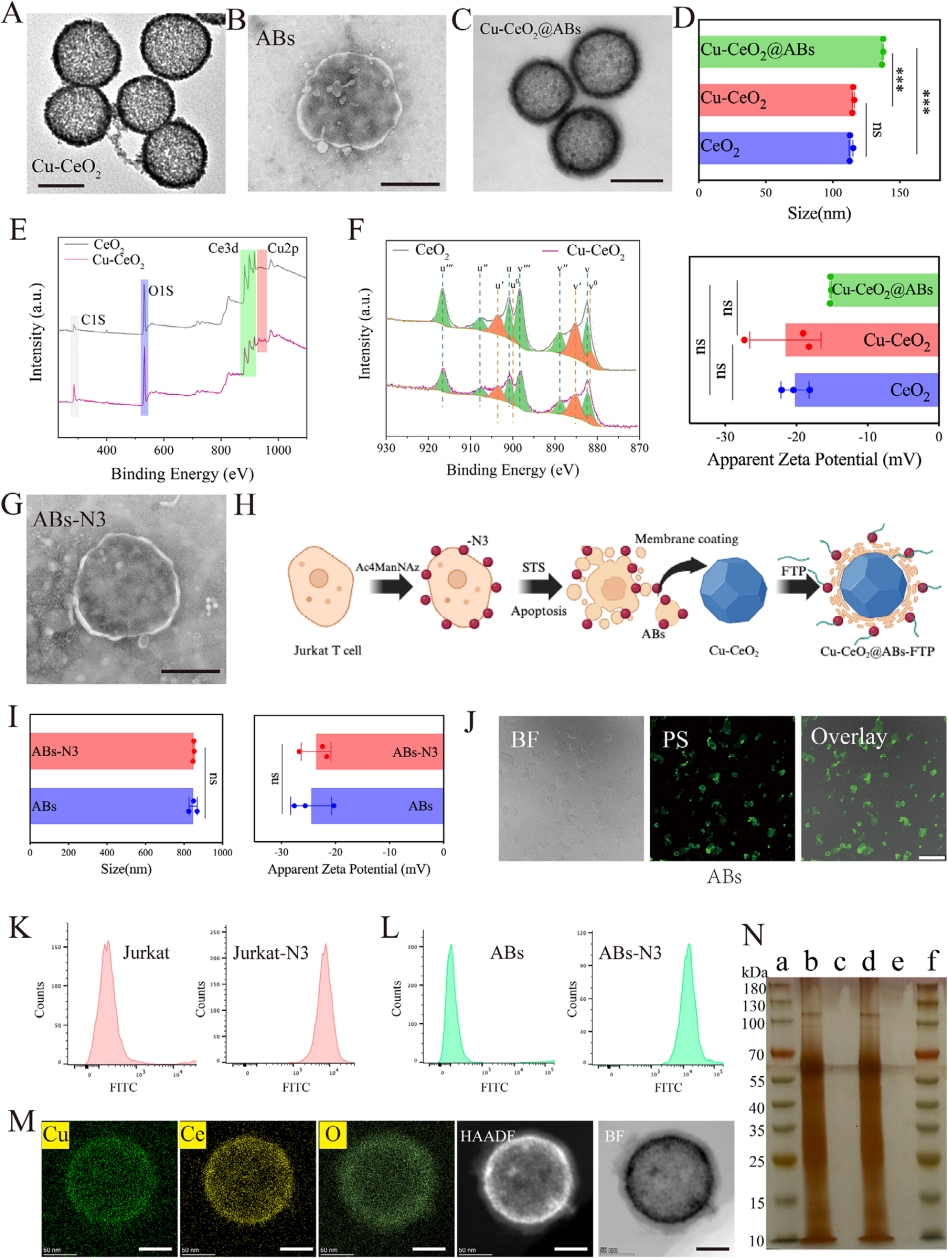
(A) TEM morphology characterization of Cu‐CeO_2_ nanoparticles, scale bar = 100 nm. (B) TEM morphology characterization of ABs, scale bar = 500 nm. (C) TEM morphology characterization of Cu‐CeO_2_@ABs, scale bar = 100 nm. (D) DLS characterization of the size and zeta potential of CeO_2_, Cu‐CeO_2,_ and Cu‐CeO_2_@ABs (n = 3). (E) XPS survey spectra of CeO_2_ and Cu‐CeO_2_. (F) XPS Ce 3d spectra of CeO_2_ and Cu‐CeO_2_. (G) TEM morphology characterization of ABs‐N_3_, scale bar = 500 nm. (H) Schematic illustration of the preparation of Jurkat T cell‐derived ABs and the synthesis of Cu‐CeO_2_@ABs‐FTP nanoparticles. (I) DLS characterization of the size and zeta potential of ABs and ABs‐N_3_ (n = 3). (J) Bright‐field and fluorescence imaging of ABs, PS labeled with Annexin V‐FITC, scale bar = 50 µm. (K) Flow cytometry analysis of Jurkat and Jurkat‐N_3_. (L) Flow cytometry analysis of ABs and ABs‐N3. (M) STEM‐EDS elemental distribution (Cu, Ce, O) and morphology characterization of Cu‐CeO_2_ nanoparticles, scale bar = 50 nm. (N) SDS‐PAGE analysis of membrane retention proteins (a: marker, b: ABs membrane, c: CeO_2_, d: Cu‐CeO_2_@ABs, e: Cu‐CeO_2_, f: marker). Data are means ± SD. ^*^ *p* < 0.05, ^**^ *p* < 0.01, ^***^ *p* < 0.001, ns, *p* ≥ 0.05.

Then, Staurosporine (STS) was used to induce apoptosis of Jurkat T cells, and ABs were extracted. Transmission Electron Microscopy (TEM) observation of ABs is shown in Figure 1B. Subsequently, Cu‐CeO_2_ and ABs were repeatedly extruded using a liposome extruder (Figure S4), ultimately yielding Cu‐CeO_2_@ABs. TEM images reveal a clear membrane structure wrapping the surface of the nanoparticles (Figure 1C). Dynamic Light Scattering (DLS) results showed that the average particle sizes of CeO_2_, Cu‐CeO_2_, and Cu‐CeO_2_@ABs were 113.62, 115.30, and 137.33 nm, respectively, and the corresponding zeta potentials were −20.21, −21.54, and −15.22 mV. After ABs membrane coating, the surface potential is slightly increased, and the particle size increases by approximately 20 nm, preliminarily indicating successful membrane coating (Figure 1D and Figure S5). The XRD pattern exhibited characteristic diffraction peaks of CeO_2_, indicating good crystallinity and high phase purity of the prepared CeO_2_ (Figure S6). The XPS survey spectra clearly showed characteristic peaks of C1s, O1s, and Ce3d in both CeO_2_ and Cu‐CeO_2_ samples, and Cu 2p‐related signals appeared in the Cu‐CeO_2_ sample, indicating that Cu was successfully introduced into the material system [24] (Figure 1E). The high‐resolution XPS spectrum of Cu 2p showed distinct Cu 2p_3/2_ and Cu 2p_1/2_ characteristic peaks at approximately 932 and 952 eV, respectively, further confirming the successful formation of Cu‐CeO_2_ (Figure S7). In addition, comparison of the high‐resolution Ce 3d XPS spectra showed that Cu‐CeO_2_ exhibited obvious coexistence peaks of Ce^3^^+^/Ce^4^^+^, with a Ce^3^^+^ content of approximately 38%, which was slightly higher than that of CeO_2_, suggesting that Cu incorporation effectively regulated the valence state of cerium and enhanced the level of oxygen vacancies (Figure 1F).

Subsequently, Jurkat T cells were treated with Ac4ManNAz to introduce N_3_ onto the cell surface, yielding Jurkat T‐N_3_ [29]. Jurkat T‐N_3_ apoptosis was then induced by STS to obtain ABs with surface N_3_ modification (ABs‐N_3_) (Figure 1G) [30]. Since one end of FTP is modified with Dibenzocyclooctyne (DBCO), which can undergo a click chemistry coupling reaction with N_3_ [31], the obtained DBCO‐FTP was reacted with Cu‐CeO_2_@ABs‐N_3_ to finally produce Cu‐CeO_2_@ABs‐FTP (Figure 1H). ABs are typically defined as vesicles with diameters ranging from 100 nm to 5 µm that are released during apoptosis [32]. DLS results show that the average particle sizes of ABs and ABs‐N_3_ are 847.29 and 849.81 nm, respectively, with zeta potentials of −24.50 and −23.57 mV, indicating that azide modification has no obvious change on the particle size or surface charge of ABs (Figure 1I and Figure S8). To further characterize ABs, ABs were incubated with an Annexin V fluorescent probe that specifically recognizes phosphatidylserine (PS) [33], and the colocalization of bright‐field (BF) images and PS fluorescence signals was compared. The results confirmed that the PS signal originated from ABs (Figure 1J). It should be noted that Annexin V–FITC staining was used only to verify that the isolated vesicles exhibited the characteristic phosphatidylserine (PS) membrane features of ABs; it does not indicate that all PS molecules on the final Cu‐CeO_2_@ABs‐FTP surface remained outward‐facing. Because a small degree of heterogeneous membrane orientation may occur during extrusion, the membrane orientation in this study was interpreted primarily on the basis of subsequent surface modification and functional validation. In addition, flow cytometry analysis demonstrated that both the treated Jurkat T cells and their derived ABs were successfully introduced with N_3_, resulting in engineered Jurkat T‐N_3_ and ABs‐N_3_ (Figure 1K,L). Finally, NPs was characterized by Scanning Transmission Electron Microscopy (STEM). Elemental mapping showed uniform distributions of Ce, O, and Cu, and the high‐angle annular dark‐field STEM (HAADF‐STEM) and BF‐STEM images revealed nanoparticles and membrane structures with regular morphology and well‐defined boundaries (Figure 1M). Furthermore, sodium dodecyl sulfate‐polyacrylamide gel electrophoresis (SDS‐PAGE) was used to analyze the total protein profiles of ABs, CeO_2_, Cu‐CeO_2_@ABs, and Cu‐CeO_2_. As shown in Figure 1N, silver staining revealed that nanoparticles coated with ABs membrane retained protein bands highly similar to those of ABs membrane, whereas uncoated inorganic nanoparticles exhibited almost no detectable protein bands, indicating that the ABs membrane was successfully coated onto the nanoparticles surface while preserving its characteristic protein composition.

These results demonstrate that CeO_2_ and Cu‐CeO_2_ nanoparticles were successfully synthesized in this study, and ABs were extracted from Jurkat T cells to achieve membrane coating of the nanoparticles, resulting in the formation of Cu‐CeO_2_@ABs. Furthermore, through the assembly of FTP, Cu‐CeO_2_@ABs‐FTP nanoparticles were constructed.

### 2.2 In Vitro Biological Functions of Nanoparticles and Targeting Ability of Cu‐CeO_2_@ABs‐FTP Nanoparticles Toward Myofibroblasts

The level of ROS in the wound microenvironment is one of the key factors influencing wound healing [34]. In this study, the catalytic activities of CeO_2_ and Cu‐CeO_2_ in multiple ROS‐related reactions were first evaluated. The results showed that, with increasing nanoparticle concentration, the scavenging efficiencies toward superoxide anion (O_2_•^−^) and hydroxyl radical (•OH) were significantly enhanced, and the overall performance of Cu‐CeO_2_ was consistently superior to that of CeO_2_, indicating that the introduction of Cu effectively enhanced the free radical scavenging capacity of the nanoparticles (Figures S9 and S10). In hydrogen peroxide (H_2_O_2_) scavenging assays, Cu‐CeO_2_ also exhibited stronger catalytic activity, enabling more efficient decomposition of H_2_O_2_ and thereby significantly reducing oxidative levels in the system [35] (Figure S11). Further oxygen generation experiments demonstrated that Cu‐CeO_2_ could continuously and efficiently generate O_2_ during the reaction process, with both the generation rate and final yield being markedly higher than those of CeO_2_, indicating enhanced catalase‐like activity (Figure S12). Taken together, the introduction of Cu significantly improved the redox homeostasis regulatory capability of CeO_2_, allowing Cu‐CeO_2_ to exhibit superior catalytic and antioxidant performance in scavenging multiple ROS and alleviating hypoxic microenvironments, thereby providing an important physicochemical basis for its mediated anti‐inflammatory and tissue repair effects [36].

To determine the optimal working concentration of the FTP‐complex for preparing Cu‐CeO_2_@ABs‐FTP, Cu‐CeO_2_@ABs‐N_3_ was reacted with 0, 20, 40, 80, and 100 µg mL^−1^ of FTP‐complex, and the coupling between the FTP‐complex and the nanoparticles was achieved through a click chemistry reaction between DBCO and N_3_ (Figure 2A). The results showed that when the concentration of FTP‐complex exceeded 20 µg mL^−1^, the relative fluorescence intensity of the supernatant increased with increasing FTP‐complex concentration, indicating that the binding efficiency between them was as high as 20 µg mL^−1^ (Figure 2B). It should be noted that DBCO‐FTP was introduced after the formation of N_3_‐modified Cu‐CeO_2_@ABs and therefore did not undergo the membrane coating and extrusion processes, avoiding potential effects caused by changes in membrane orientation. Although the possibility of minor membrane orientation heterogeneity cannot be completely excluded, FTP is expected to primarily conjugate with surface‐accessible N_3_ sites. The successful construction of Cu‐CeO_2_@ABs‐FTP and its selective binding to FAP‐high‐expressing myofibroblasts demonstrate that FTP retains its functional activity on the final particle surface. Further flow cytometry analysis demonstrated that, with increased FTP‐complex concentration, its binding ability to myofibroblasts exhibited a dose‐dependent trend and gradually reached a plateau at 20 µg mL^−1^ (Figure 2C). Therefore, 20 µg mL^−1^ was selected as the optimal concentration for the subsequent experiments.

**FIGURE 2:**
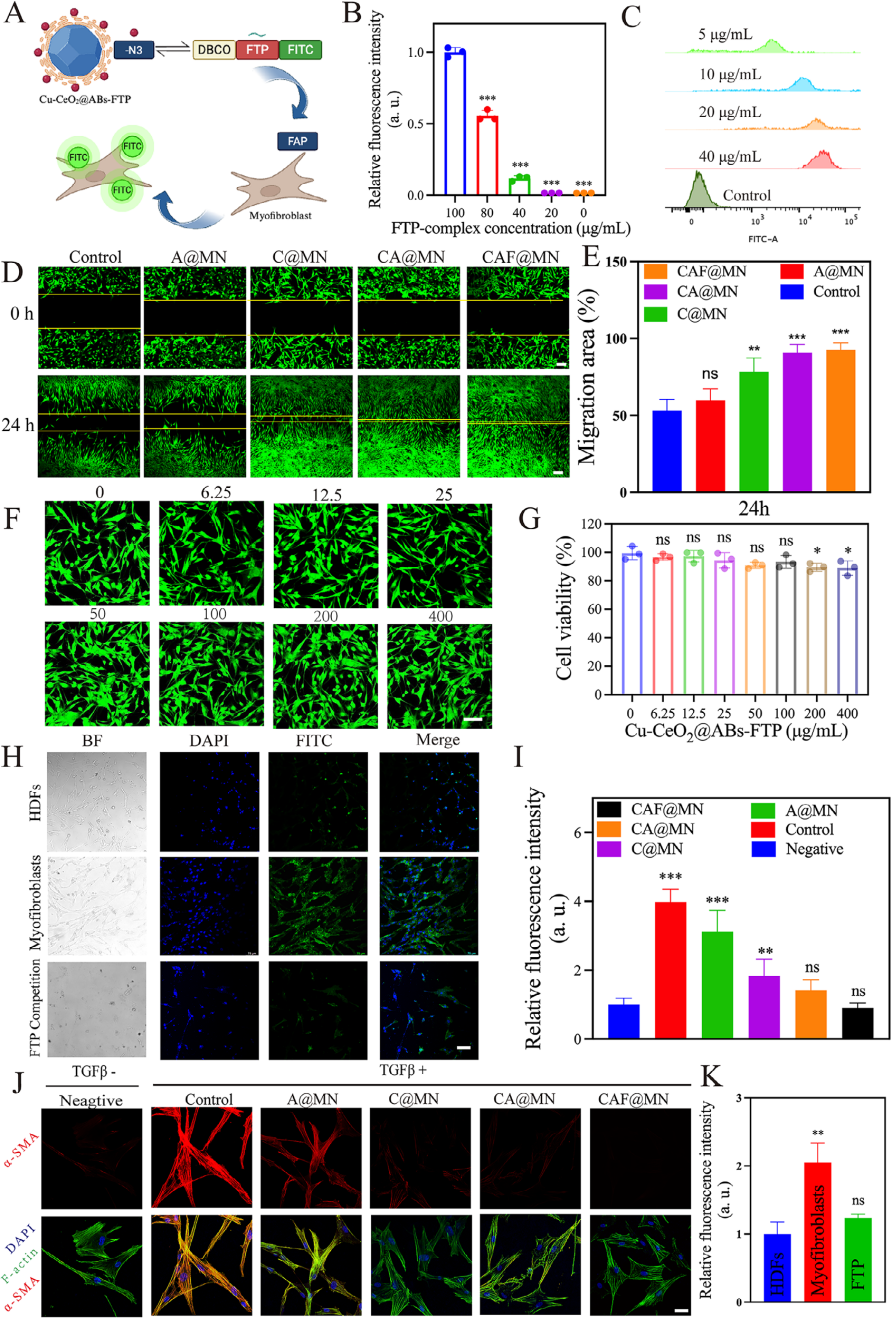
(A) Schematic illustration of the conjugation between Cu‐CeO_2_@ABs nanoparticles and FTP via azide‐DBCO coupling, and the targeted recognition of FAP‐overexpressing myofibroblasts by Cu‐CeO_2_@ABs‐FTP. (B) Relative fluorescence intensity at different FTP‐complex concentrations (n = 3). (C) Flow cytometry analysis of the binding ability to myofibroblasts at different FTP‐complex concentrations. (D) Effects of different treatments on human dermal fibroblasts (HDFs) migration, observed at 0 and 24 h after wound healing assay, scale bar = 100 µm. (E) Quantitative analysis of HDFs migration under different treatment groups (n = 3). (F) Live/dead staining analysis of HDFs after being treated by different working concentration of the nanoparticles (µg mL^−1^), scale bar = 100 µm. (G) CCK‐8 quantitative analysis of cell viability after treatment with Cu‐CeO_2_@ABs‐FTP at different concentrations (n = 3). (H) Confocal fluorescence images of HDFs, myofibroblasts, and the free FTP competition group. BF indicates bright‐field images; nuclei are stained with DAPI (blue), and Cu‐CeO_2_@ABs‐FTP labeled with FITC is shown in green, scale bar = 75 µm. (I) Quantitative analysis of α‐SMA (red) fluorescence intensity in panel J (n = 3). (J) Immunofluorescence staining shows TGF‐β‐induced myofibroblast phenotypic changes of HDFs under different treatment conditions, with α‐SMA shown in red, F‐actin in green, and nuclei (DAPI) in blue, scale bar = 25 µm. (K) Quantitative analysis of FITC fluorescence intensity in panel H (n = 3). Data are means ± SD. ^*^ *p* < 0.05, ^**^ *p* < 0.01, ^***^ *p* < 0.001, ns, *p* ≥ 0.05.

To systematically evaluate the functions and biosafety of different nanoparticles, serial of microneedle patches composed of ABs membrane (A@MN), Cu‐CeO_2_ (C@MN), Cu‐CeO_2_@ABs (CA@MN), and Cu‐CeO_2_@ABs‐FTP (CAF@MN) were respectively prepared using HAMA hydrogel as the matrix. Conditioned media (CM) from different groups were collected to treat human dermal fibroblasts (HDFs). The cell scratch assay results showed that after 24 h of culture, compared with the Control group, all treatment groups promoted the migration of HDFs toward the scratch area, with particularly pronounced effects observed in the CA@MN and CAF@MN groups (Figure 2D,E). In addition, to verify the effects of nanoparticles on angiogenesis‐related behaviors, cell scratch assays were performed on human umbilical vein endothelial cells (HUVECs). The results demonstrated that after 24 h of culture, compared with the Control group, all experimental groups promoted HUVECs migration toward the scratch area to varying degrees, among which the CAF@MN group exhibited the most significant scratch closure effect, with a 24 h cell migration rate of approximately 95.17% (Figures S13 and S14). These results suggest that CAF@MN has a marked advantage in enhancing endothelial cell migration, which may be attributed to the loaded Cu‐CeO_2_ reducing excessive ROS levels and promoting nitric oxide (NO) generation, thereby activating signaling pathways associated with endothelial cell migration and angiogenesis [37]. To further validate the pro‐angiogenic potential of CAF@MN at the functional level, tube formation assays were performed based on HUVEC. It was found that the Control group formed only a limited number of sparse and discontinuous tubular structures, while A@MN exhibited a relatively modest improvement. In contrast, the C@MN, CA@MN, and CAF@MN groups all promoted the formation of vascular‐like networks to varying degrees. Notably, the CA@MN and CAF@MN groups exhibited more intact and continuous tubular structures. Quantitative analysis revealed that the total branch length, number of nodes, and number of junctions were significantly increased in the CA@MN and CAF@MN groups, with the CAF@MN group demonstrating the most prominent pro‐angiogenic effects across all three parameters (Figure S15). These findings indicate that CAF@MN effectively enhances the tube formation capacity of HUVECs, further supporting its potential to promote angiogenesis. To further evaluate the in vitro biosafety of Cu‐CeO_2_@ABs‐FTP, HDFs were treated with gradient concentrations of 0, 6.25, 12.5, 25, 50, 100, 200, and 400 µg mL^−1^. Live/dead staining results showed that cells in all groups were predominantly viable with intact morphology, and almost no obvious dead cell signals were observed (Figure 2F). CCK‐8 assay results further indicated that within the concentration range of 6.25–400 µg mL^−1^, HDFs maintained high cell viability, with no significant cytotoxic responses compared with the control group (0 µg mL^−1^) (Figure 2G). Taken together, these results demonstrate that this nanoparticle system exhibits no obvious cytotoxicity within the concentration range of 0–400 µg mL^−1^ and possesses good in vitro biosafety.

Furthermore, to evaluate the targeting ability and specificity of Cu‐CeO_2_@ABs‐FTP toward myofibroblasts, FITC‐labeled Cu‐CeO_2_@ABs‐FTP was used to treat HDFs, myofibroblasts, and myofibroblasts pre‐incubated with an excess amount of free FTP, respectively. Confocal microscopy revealed only weak FITC fluorescence signals in HDFs, whereas myofibroblasts exhibited markedly enhanced fluorescence intensity, indicating a stronger binding capability of Cu‐CeO_2_@ABs‐FTP toward myofibroblasts. Notably, pre‐incubation with excess free FTP significantly reduced the fluorescence intensity in myofibroblasts, approaching the level observed in the HDFs group, suggesting that free FTP competitively inhibited the binding of Cu‐CeO_2_@ABs‐FTP to myofibroblasts (Figure 2H,K). These results further demonstrate that FTP functionalization endows the nanoparticles with efficient myofibroblast‐specific targeting capability. This phenomenon may be closely associated with the high‐level expression of FAP in myofibroblasts; during the middle and late stages of wound healing, excessive activation of myofibroblasts often leads to scar hyperplasia [38]. Therefore, the selective binding ability of the nanoparticles toward fibroblasts and myofibroblasts is beneficial for the precise identification of functionally overactive myofibroblasts, thereby achieving anti‐fibrotic effects and inhibition of hypertrophic scar. In addition, to verify the feasibility of the anti‐fibrotic effect of Cu‐CeO_2_@ABs‐FTP, the fluorescence expression level of the myofibroblast marker α‐SMA was evaluated in vitro [39]. Under TGF‐β induction, different groups of CM were added for treatment [40]. Compared with the control group, the expression of α‐SMA in myofibroblasts was significantly reduced in the C@MN, CA@MN, and CAF@MN groups, with the most pronounced inhibitory effect observed in the CAF@MN group, demonstrating favorable in vitro anti‐fibrotic potential (Figure 2I,J).

Taken together, Cu‐CeO_2_@ABs‐FTP nanoparticles exhibited good in vitro biosafety and promoted the migration and maintained the viability of HDFs and HUVECs. Meanwhile, they showed stronger binding affinity toward myofibroblasts and effectively inhibited the fibrotic process, which is beneficial for targeted recognition and suppression of excessive myofibroblast activation during the middle and late stages of wound healing, thereby preventing hypertrophic scar.

### 2.3 Synthesis and Characterization of CAF@MN

Microneedles can penetrate the skin barrier in a minimally invasive manner, enabling precise and sustained delivery of nanoparticles, thereby accelerating wound healing and reducing inflammation and hypertrophic scar [41]. First, 5% (w/v) HAMA hydrogel containing 100 µg mL^−1^ Cu‐CeO_2_@ABs‐FTP nanoparticles was added dropwise into the microneedle mold, and air bubbles were removed under vacuum. After drying, poly (vinyl alcohol) (PVA) was added, followed by ultraviolet light curing to form a composite structure. The solidified microneedles were then demolded to obtain the CAF@MN, which was applied to the wound site for localized delivery and treatment (Figure 3A and Figure S16). Figure 3B shows the Scanning Electron Microscopy (SEM) morphology of CAF@MN, revealing intact microneedle structures with a regular conical shape, dense surfaces, and clear contours, with a needle height of approximately 350 µm and a base diameter of approximately 200 µm. SEM‐Energy Dispersive X‐ray Spectroscopy (EDS) elemental mapping further demonstrated uniform distributions of Ce, Cu, and O within the microneedle structure, indicating that the nanoparticles were successfully and stably loaded into the microneedle system.

**FIGURE 3:**
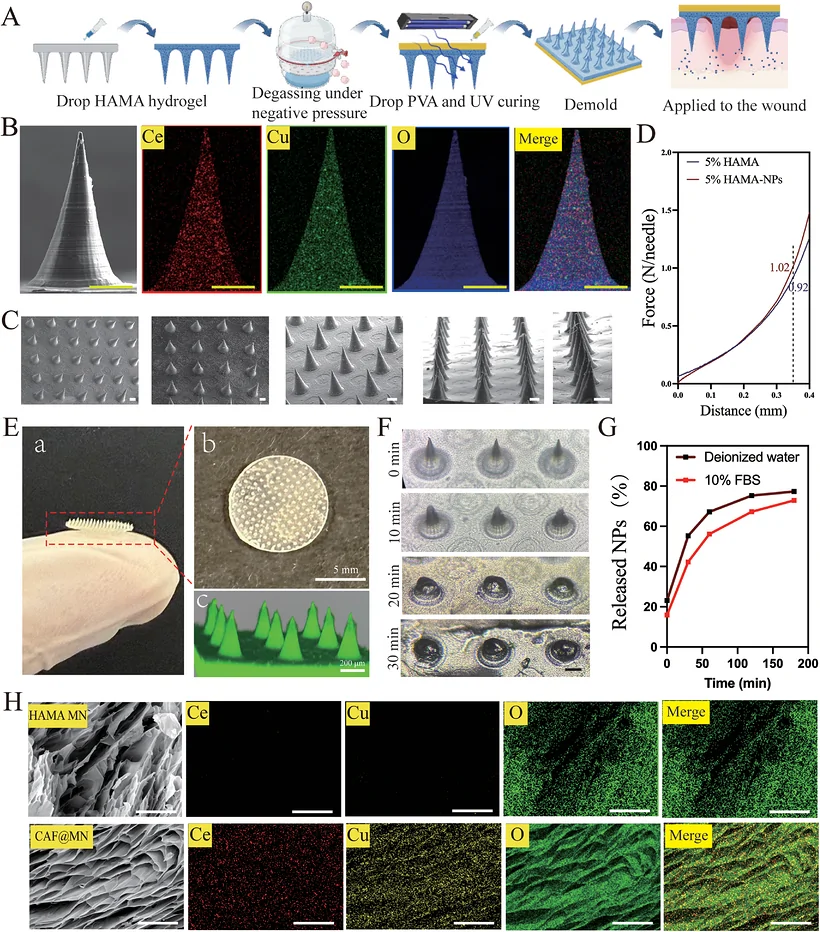
(A) Schematic illustration of the fabrication of nanoparticle‐loaded HAMA hydrogel microneedle patches and the release of nanoparticles after application to skin tissue. (B) SEM images of CAF@MN and elemental mapping of Ce, O, and Cu within the microneedle structure, scale bar = 100 µm. (C) SEM images of the CAF@MN array from different angles show a well‐ordered and structurally intact architecture, scale bar = 100 µm. (D) Force‐displacement curves of MN‐5% HAMA and CAF@MN‐5% HAMA (100 µg mL^−1^). (E) Photographs of CAF@MN (a, b) and confocal microscopy images (c) show its overall morphology and the distribution of FITC within the array; scale bars are indicated in the images. (F) Morphological images of CAF@MN after different moisture absorption times at 75% humidity and room temperature, scale bar = 100 µm. (G) Cumulative release profiles of nanoparticles from CAF@MN in deionized water and PBS containing 10% (v/v) FBS. (H) Microstructural features and distributions of Ce, Cu, and O in HAMA MN and CAF@MN, scale bar = 100 µm.

To further comprehensively characterize the microneedle morphology, Figure 3C presents SEM images of CAF@MN at different magnifications and observation angles. The results show that the microneedle arrays are regularly arranged, uniform in size, and well formed. Side‐view and high‐magnification images further confirm that the microneedles exhibit a clear conical structure and good structural consistency (Figure 3E(a,b)). The microneedles and substrate were labeled with FITC and observed under confocal microscopy, revealing microneedle and substrate structures with regular overall morphology and uniform fluorescence distribution (Figure 3E(c)). Further confocal *Z*‐axis scanning was performed to observe the cross‐sectional structure of the microneedles. Sequential scanning along the vertical direction from the substrate and needle base to the needle tip showed that the microneedle array structure was intact and regularly arranged, with continuous fluorescence distribution along the height direction, indicating good three‐dimensional structural integrity. This provides a reliable structural basis for effective skin penetration and stable delivery (Figures S17 and S18). In addition, to verify the skin penetration capability of the microneedles, a universal testing machine was used to perform compressive mechanical tests on 5% (w/v) HAMA hydrogel microneedles and 5% (w/v) HAMA hydrogel microneedles containing 100 µg mL^−1^ Cu‐CeO_2_@ABs‐FTP nanoparticles [42] (Figure S19). The force‐distance curves showed that both groups exhibited good mechanical strength and structural stability during compression, with the compressive force of the microneedles at full‐tip‐height deformation of 0.92 and 1.02 N/needle, respectively, which were markedly higher than the critical threshold required to puncture the stratum corneum (0.045 N), demonstrating that CAF@MN possesses a mechanical basis for effective skin penetration and delivery [22, 43] (Figure 3D).

In addition, the hygroscopic property of CAF@MN was evaluated to characterize its stability [43]. CAF@MN patches were placed in an environment with a relative humidity of 75%, and morphological changes were recorded at 0, 10, 20, and 30 min. The results showed that CAF@MN and its microneedle tips gradually absorbed moisture under humid conditions and underwent obvious dissolution after 30 min, indicating good hygroscopic property (Figure 3F and Figure S20). This property is favorable for the rapid release of nanoparticles from the microneedles within a short period of time, allowing the released nanoparticles to continuously exert their functions during wound healing, while also rapidly absorbing wound exudates, which is beneficial for the treatment of exudative wounds [44]. To further investigate the release efficiency of nanoparticles from the microneedles, Figure 3G shows the cumulative release behavior of nanoparticles from CAF@MN in deionized water over time (initial concentration: 100 µg mL^−1^). The results showed that the release rate increased rapidly at the early stage and then gradually reached a plateau, with a cumulative release approaching 80% at 150 min, demonstrating that CAF@MN exhibits high and stable release efficiency. To further evaluate the release behavior of CAF@MN under conditions closer to the wound microenvironment, PBS containing 10% (v/v) FBS was used as a protein‐containing release medium. The results showed that CAF@MN still exhibited an initial rapid release phase followed by a gradual plateau in this medium, although the overall release rate was slightly lower than that observed in the deionized water group. At 150 min, the cumulative release rate in the PBS containing 10% FBS group reached approximately 70%–75%, indicating that proteins and physiological ionic conditions could partially delay nanoparticle release. Nevertheless, CAF@MN maintained a sustained and effective release profile under these conditions (Figure 3G). Figure 3H shows the microscopic morphology and elemental distribution characteristics of HAMA MN and CAF@MN. SEM images revealed that HAMA MN exhibited a non‐uniform pore size distribution, and no obvious Ce or Cu signals were detected in the SEM‐EDS elemental mapping. In contrast, after the introduction of Cu‐CeO_2_@ABs‐FTP nanoparticles, CAF@MN displayed a more uniform pore size distribution, and SEM‐EDS elemental mapping showed uniform distribution of Ce and Cu elements within the hydrogel. These results indicate that Cu‐CeO_2_@ABs‐FTP nanoparticles were successfully introduced and stably dispersed in the HAMA hydrogel system. Their incorporation not only facilitates uniform distribution and controllable release of the nanoparticles but may also enhance the overall structural stability and mechanical properties of the hydrogel microneedles by optimizing the internal pore structure [45].

### 2.4 Antibacterial Effects of CAF@MN and Targeting Ability of Nanoparticles Toward Macrophages

Methicillin‐resistant *Staphylococcus aureus* (MRSA) and *Escherichia coli* (*E. coli*) are common pathogens in wound infections. In the weakly acidic and highly oxidative stress microenvironment shaped by bacterial infection, CeO_2_ has been demonstrated to participate in reactions via surface vacancies, catalyzing the generation of ROS to disrupt bacterial membrane integrity. The introduction of Cu further enhances the antibacterial efficiency of this system: copper ions can interact with thiol and amino groups on bacterial cell membranes, destabilizing membrane structures and interfering with the normal functions of key enzymes; meanwhile, Cu‐involved redox reactions can accelerate the accumulation of endogenous ROS in bacteria, thereby inducing irreversible bacterial damage. Through the synergistic bactericidal properties of Cu^+^/Cu^2+^ and the redox characteristics of Ce^3+^/Ce^4+^, bacterial cell membrane integrity is disrupted and bacterial proteins and DNA are damaged, resulting in efficient broad‐spectrum antibacterial activity [46, 47]. Figure 4A shows the antibacterial effects of different treatment groups against MRSA and *E. coli*. Plate culture results demonstrated that, compared with the Control and A@MN groups, the numbers of surviving colonies in the C@MN, CA@MN, and CAF@MN groups were significantly reduced, with markedly decreased bacterial survival rates, indicating stronger antibacterial activity (Figure 4C,D). In addition, Figure 4B presents SEM observations of bacterial morphological changes in each group. The results showed that MRSA and *E. coli* in the Control group exhibited intact morphology with smooth surfaces, whereas bacteria treated with the experimental groups displayed obvious cell wall damage, collapse, structural disintegration, and leakage of intracellular contents. Among them, the CAF@MN group exhibited the most pronounced antibacterial effects against both MRSA and *E. coli* (Figure 4E,F).

**FIGURE 4:**
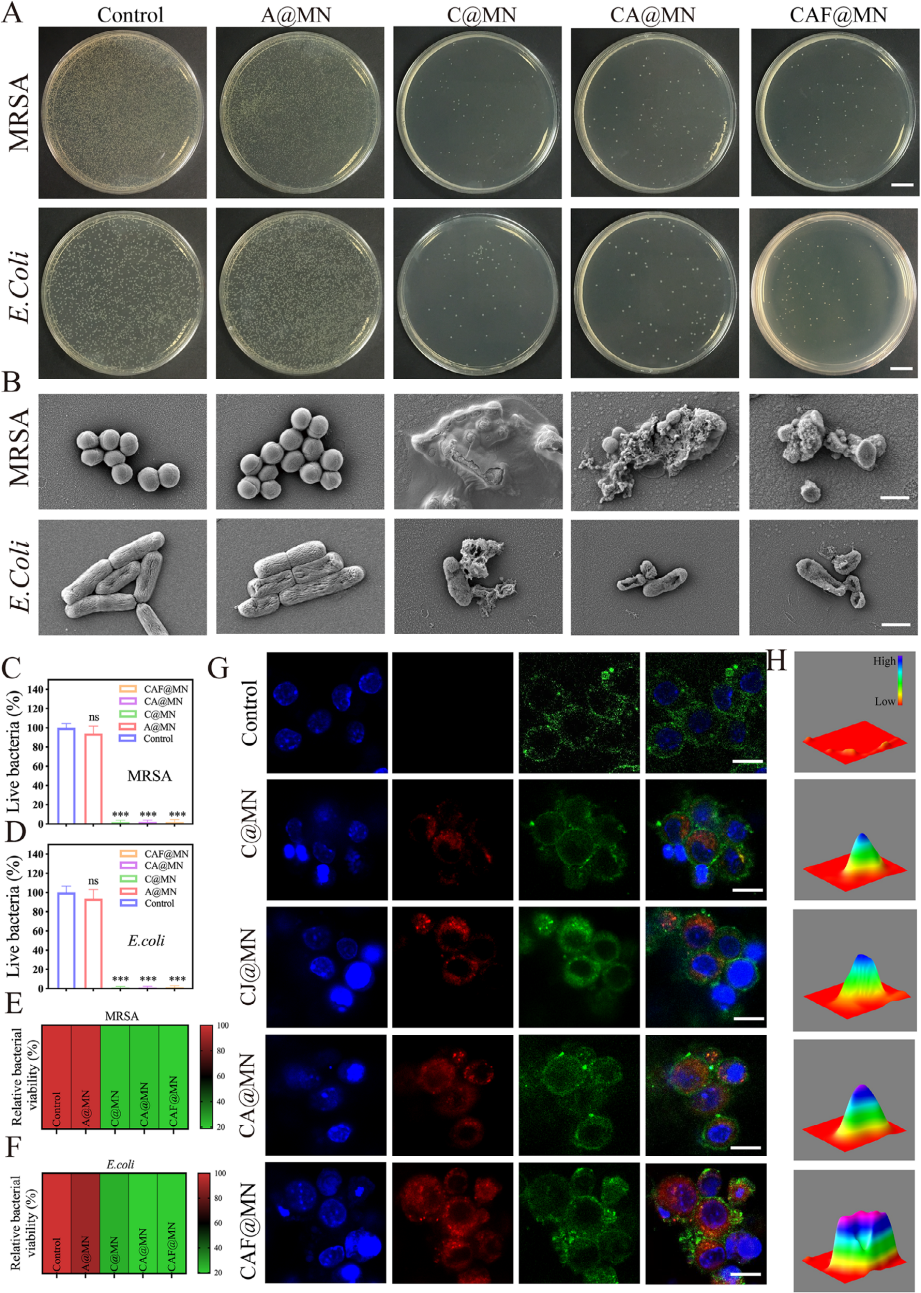
(A) Images of surviving Methicillin‐resistant Staphylococcus aureus (MRSA) and *Escherichia coli* (*E. coli*) colonies on agar plates under different treatment groups, scale bar = 1 cm. (B) SEM images of MRSA and *E. coli* show morphological changes of bacteria after different treatments, scale bar = 1 µm. (C, D) Quantitative analysis of the relative survival rates of MRSA (C) and *E. coli* (D) in different treatment groups, along with the corresponding bacterial viability heatmap analyses of MRSA (E) and *E. coli* (F) (n = 3). (G) Confocal imaging shows the endocytosis of different nanoparticles by macrophages, in which Hoechst 33342 (blue) labels the nuclei, DiO (green) labels the cell membrane, and Cy5.5 (red) labels the nanoparticles. Scale bar = 10 µm. (H) 3D surface plot visualization of panel G. Data are means ± SD. ^*^ *p* < 0.05, ^**^ *p* < 0.01, ^***^ *p* < 0.001, ns, *p* ≥ 0.05.

To further evaluate the effect of CAF@MN on bacterial biofilms, crystal violet staining was performed to assess the disruption capability of different treatments against biofilms of MRSA and E. coli. Compared with the Control group, the A@MN group showed no significant changes in biofilm staining or relative biofilm biomass, whereas the C@MN, CA@MN, and CAF@MN groups exhibited markedly reduced crystal violet staining intensity and significantly decreased relative biofilm biomass. Among them, the CAF@MN group demonstrated the lowest residual biofilm biomass and the strongest biofilm‐disrupting effect against both bacterial species (Figure S21A–C). These results indicate that CAF@MN not only inhibits the growth of planktonic bacteria but also effectively reduces bacterial biofilms, further supporting its potential application in the treatment of infected wounds.

Inflammatory responses are also a major challenge that must be overcome during wound healing. Enhancing the targeting ability of nanoparticles toward inflammatory regions is beneficial for inhibiting M1 polarization of macrophages and promoting anti‐inflammatory wound repair [48]. In this study, RAW 264.7 macrophages were first treated with different nanoparticles at a concentration of 100 µg mL^−1^, all of which were labeled with Cy5.5, and their uptake by macrophages was observed after 6–8 h of incubation. Confocal microscopy results showed (Figure 4G,H and Figure S22) significant differences in intracellular red fluorescence intensity among different treatment groups. Compared with the Control group, the experimental groups exhibited stronger red fluorescence accumulation within macrophages, with the CAF@MN group showing the most pronounced signal, indicating that the ABs membrane endowed the nanoparticles with effective macrophage‐targeting capability and enhanced macrophage recognition and phagocytosis of the nanoparticles. To further determine whether the macrophage‐targeting effect exhibited cell‐origin dependency, CH@MN coated with HDF cell membranes, CHA@MN coated with HDF‐derived ABs membranes, CJ@MN coated with Jurkat cell membranes, and CA@MN coated with Jurkat‐derived ABs membranes were compared in parallel. Flow cytometry analysis showed that the intracellular fluorescence signals in macrophages exhibited an overall increasing trend in the order of C@MN, CH@MN, CJ@MN, CHA@MN, and CA@MN (Figure S23A). The macrophage uptake of both ABs membrane‐coated groups was higher than that of their corresponding intact cell membrane‐coated groups, indicating that ABs membranes enhanced the recognition and phagocytosis of nanoparticles by macrophages. Furthermore, the CA@MN group exhibited stronger fluorescence signals than the HDF‐derived CHA@MN group, suggesting that Jurkat‐derived ABs possess a more pronounced cell‐origin‐dependent advantage in macrophage targeting. To further distinguish the macrophage‐targeting advantage mediated by ABs membranes from the general effects of cell membrane coating, a Jurkat cell membrane‐coated nanoparticle group was additionally included. Jurkat cell membranes were coated onto the surface of Cu‐CeO_2_ to prepare Cu‐CeO_2_@JM, which was subsequently incorporated with HAMA to fabricate MN containing Cu‐CeO_2_@JM, termed CJ@MN. Under the same treatment conditions, the macrophages in the CJ@MN group exhibited stronger Cy5.5 fluorescence signals than those in the uncoated C@MN group, but significantly weaker signals than those in the ABs membrane‐coated CA@MN group (Figure 4G,H). These results indicate that conventional Jurkat cell membrane coating can partially enhance nanoparticle uptake by macrophages, whereas ABs membranes further promote macrophage recognition and phagocytosis. This finding demonstrates that the macrophage‐targeting advantage of ABs membranes is not solely attributable to the general membrane coating effect, but may be associated with apoptosis‐related recognition signals, such as phosphatidylserine, retained on the ABs membranes.

### 2.5 In Vitro Anti‐Inflammatory and Antioxidative Effects of CAF@MN

Furthermore, to investigate the anti‐inflammatory effects exerted after nanoparticle uptake by RAW 264.7 macrophages, RAW 264.7 macrophages were first stimulated with 100 ng mL^−1^ lipopolysaccharide (LPS) for 24 h to induce M1 polarization, and RAW 264.7 macrophages were labeled with F4/80 and CD86. Flow cytometry results showed that the proportion of F4/80^+^ and CD86^+^ double‐positive cells increased from 20.10% to 62.10%, confirming the successful induction of M1 polarization in macrophages (Figure S24). Subsequently, the cells were further incubated with different groups of CM for 48 h, and CD206 was used as a marker to evaluate M2 polarization of macrophages. Immunofluorescence results showed that, compared with the control group, CD206‐positive signals in macrophages from all experimental groups were enhanced to varying degrees, indicating macrophage polarization toward the M2 phenotype. Among them, the CAF@MN group exhibited the most pronounced red fluorescence expression of CD206, suggesting a superior ability to promote M2 polarization of macrophages (Figure 5A,B). In addition, macrophages subjected to different treatments were double‐labeled with F4/80 and CD206 and analyzed by flow cytometry in parallel. The results showed significant differences in the proportion of F4/80^+^, CD206^+^ macrophages among groups; compared with the control group (11.20%), the proportion of F4/80^+^, CD206^+^ cells in the CAF@MN group was markedly increased (53.50%), further demonstrating that CAF@MN effectively promotes macrophage polarization toward the M2 phenotype (Figure 5C,H). To further compare the immunomodulatory capacities of membranes derived from different cell sources and ABs membranes, flow cytometry was performed to evaluate CD206 expression in macrophages treated with C@MN, CH@MN, CHA@MN, CJ@MN, and CA@MN. The results showed that the CD206 fluorescence intensity exhibited an overall increasing trend in the order of C@MN, CH@MN, CJ@MN, CHA@MN, and CA@MN (Figure S23B). Both CHA@MN and CA@MN induced stronger CD206 expression than their corresponding intact cell membrane‐coated groups, while the Jurkat‐derived CA@MN group exhibited the highest CD206 expression level. These results demonstrate that ABs membranes possess a stronger capacity to promote macrophage M2‐like polarization than the general cell membrane coating effect. Moreover, Jurkat‐derived ABs exhibited superior immunomodulatory activity compared with HDF‐derived ABs, supporting their cell‐origin‐dependent advantage in promoting macrophage M2‐like polarization. Real‐time quantitative polymerase chain reaction (RT‐qPCR) results similarly showed significant differences in the expression levels of M1‐ and M2‐related genes in macrophages among different treatment groups: compared with the control group, the expression of M1‐related genes CD80 and CD86 was downregulated to varying extents in the experimental groups, whereas the expression of M2‐related genes CD206 and CD163 was significantly upregulated, with the most pronounced changes observed in the CAF@MN group (Figure 5D–G). Collectively, these results indicate that all experimental groups can inhibit macrophage M1 polarization and promote polarization toward the anti‐inflammatory M2 phenotype to a certain extent, with the CAF@MN group exhibiting the strongest anti‐inflammatory regulatory capacity. This effect may be associated with the synergistic action of the ABs membrane and Cu‐CeO_2_ in regulating macrophage ROS homeostasis and activating pro‐repair signaling pathways such as PI3K‐AKT, thereby upregulating M2 markers including CD206 [49, 50].

**FIGURE 5:**
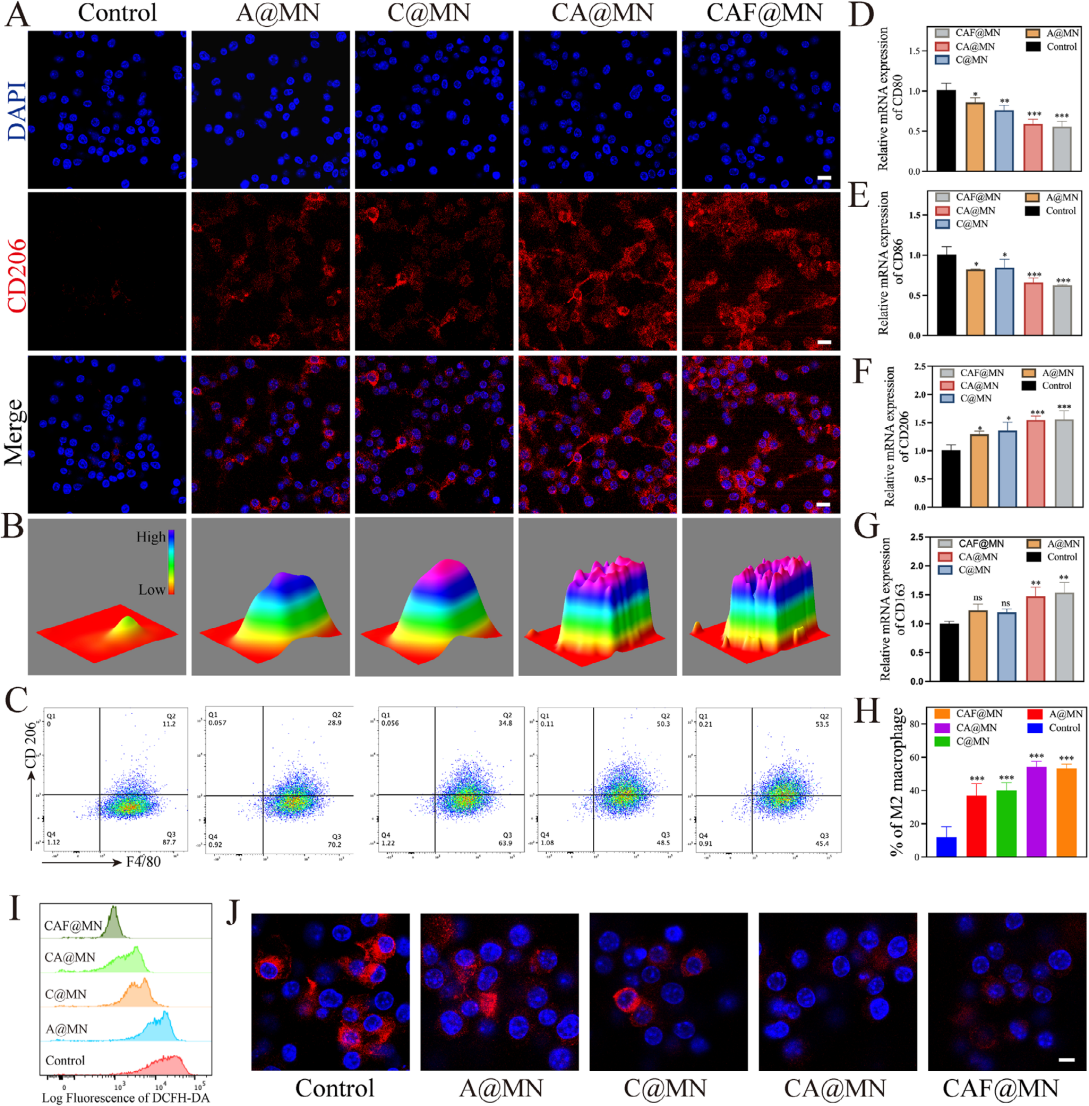
(A) Confocal microscopy images show CD206 expression in macrophages under different treatment groups, scale bar = 10 µm. (B) 3D surface plot visualization of panel A. (C) Flow cytometry analysis shows the polarization of RAW 264.7 macrophages from the M1 to M2 phenotype under different treatment groups, with macrophages labeled using F4/80 and CD206. (D–G) RT‐qPCR analysis shows changes in the relative mRNA expression levels of CD80 (D), CD86 (E), CD206 (F), and CD163 (G) in RAW 264.7 macrophages under different treatment groups (n = 3). (H) Quantitative analysis of the proportion of M2 macrophages shown in panel C (n = 3). (I) Flow cytometry analysis shows the ROS scavenging capacity of different groups. (J) Confocal microscopy shows the intracellular O_2_•^−^ levels in RAW 264.7 macrophages after treatment with different groups, scale bar = 10 µm. Data are means ± SD. ^*^ *p* < 0.05, ^**^ *p* < 0.01, ^***^ *p* < 0.001, ns, *p* ≥ 0.05.

The ability of CAF@MN to promote the transition of macrophages toward an M2‐like reparative phenotype may result from the synergistic effects of Cu‐CeO_2_ and ABs. Cu‐CeO_2_ provides a favorable microenvironment for macrophage M2‐like polarization by scavenging excessive ROS, alleviating oxidative stress, and reducing inflammatory responses. Meanwhile, previous studies have demonstrated that ABs themselves can promote inflammation resolution and the transition of macrophages toward a reparative phenotype, which may be associated with the exposure of PS on their membrane surfaces. To further verify the role of PS, we included an additional CJ@MN group coated with conventional Jurkat cell membranes and a PS‐blocked CA@MN group. Flow cytometry analysis showed that CD206 expression in the CJ@MN group was higher than that in the C@MN group but lower than that in the ABs membrane‐coated CA@MN group. After pre‐blocking PS on the surface of ABs membranes, CD206 expression in the PS‐blocked CA@MN group was significantly reduced (Figure S25). Since all groups contained the same Cu‐CeO_2_ core, these results indicate that the ability of CA@MN to promote macrophage M2‐like polarization is not only associated with Cu‐CeO_2_‐mediated ROS scavenging but also closely related to the anti‐inflammatory immunomodulatory effect mediated by PS on the surface of ABs membranes.

Since the nanoparticles exhibited good ROS scavenging capacity in vitro, their effects on alleviating intracellular endogenous ROS were further investigated. RAW 264.7 macrophages induced by LPS were treated with different groups of CM, and intracellular ROS levels were analyzed by flow cytometry using 2′,7′‐dichlorofluorescein diacetate (DCFH‐DA). The results showed that, compared with the Control group, the CAF@MN group exhibited a markedly reduced DCFH‐DA fluorescence intensity, highlighting its enhanced ROS scavenging ability (Figure 5I). This trend was further confirmed by confocal microscopy observation of intracellular O_2_•^−^ levels in RAW 264.7 macrophages (Figure 5J).

Taken together, this study systematically elucidates the comprehensive advantages of CAF@MN in regulating the wound microenvironment from multiple aspects, including antibacterial activity, immunomodulation, and anti‐oxidative regulation. Previous studies have demonstrated that both Cu‐CeO_2_ and ABs can serve as key functional components in promoting M2 polarization of macrophages [7, 51]. In this study, it was further revealed that ABs membrane derived from Jurkat T cells possess favorable macrophage‐targeting capability, and membrane coating of nanoparticles with ABs significantly enhances nanoparticle uptake by macrophages. On this basis, CAF@MN enables efficient local release of Cu‐CeO_2_@ABs‐FTP nanoparticles via microneedle‐mediated delivery, facilitating their uptake by macrophages and subsequent functional effects. The enhanced antioxidant catalytic activity mediated by Cu‐CeO_2_ effectively alleviates ROS accumulation and hypoxic conditions in the inflammatory microenvironment, while the synergistic effect of the ABs membrane further amplifies its immunomodulatory function, thereby suppressing the expression of pro‐inflammatory genes in M1 macrophages and markedly upregulating the expression of M2‐associated anti‐inflammatory genes. Through the coordination of these multiple mechanisms, macrophages are ultimately driven toward a reparative M2 phenotype, effectively inhibiting wound inflammation and providing favorable conditions for rapid, orderly, and high‐quality wound healing.

### 2.6 CAF@MN Promotes Mouse Whole‐Phase Skin Wound Healing and Tissue Regeneration

Based on the above in vitro results, including the evaluation of in vitro biosafety in representative cells, verification of anti‐inflammatory and antibacterial properties, and in vitro regulatory effects related to anti‐fibrosis, this study further established a whole‐phase skin wound healing model using 6–8 week old BALB/c mice and systematically evaluated skin tissue regeneration under different treatments [10]. Briefly, circular full‐thickness skin defects with a radius of 5 mm were created on the dorsal skin of mice, and the wounds were treated with A@MN, C@MN, CA@MN, and CAF@MN, respectively. Medical fixation film was then applied to secure the microneedle patches and prevent detachment during free movement of the mice (Figure S26). Wound healing was continuously observed and photographed on postoperative days 0, 3, 7, and 14. The results showed that wounds in the Control group were not completely healed at the end of the observation period, with obvious residual wounds and local redness and swelling. In contrast, the CAF@MN group exhibited a significantly accelerated wound closure rate, with complete wound healing observed on day 14, no apparent redness, swelling, or scar tissue formation, and obvious hair regrowth, demonstrating the most favorable healing effect (Figure 6A,B).

**FIGURE 6:**
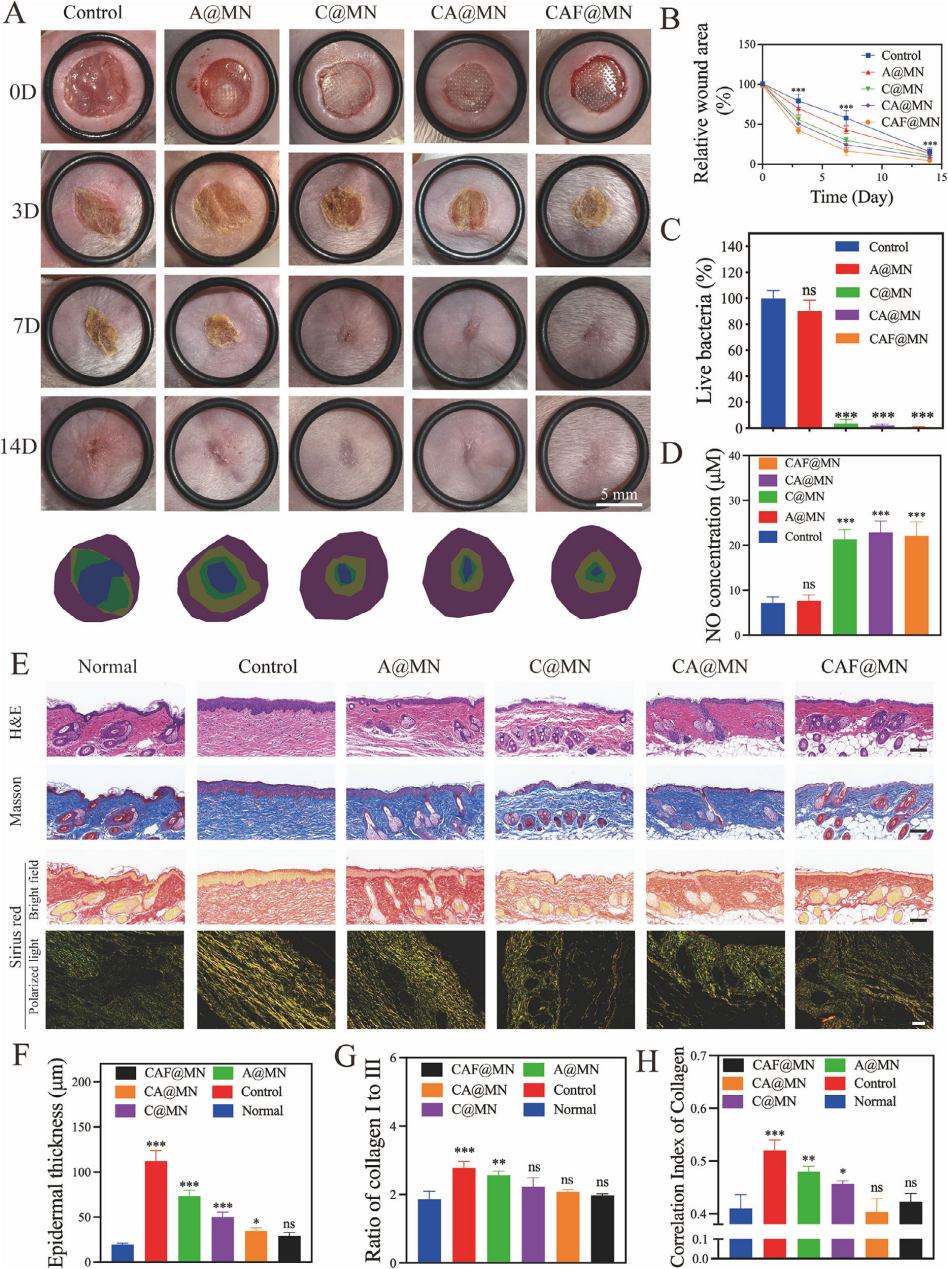
(A) Photographs of wounds of BALB/c mice under different treatments at different time points; scale bar = 5 mm. (B) Quantitative analysis of the relative wound areas at different times (n = 3). (C) Evaluation of the antibacterial efficacy of different treatment groups in vivo in mice (n = 3). (D) Comparison of nitric oxide (NO) levels in wound tissues of mice from different treatment groups (n = 3). (E) Representative images of H&E staining, Masson's trichrome staining, and Sirius red staining (bright‐field and polarized light) of wound tissues on day 14. Scale bar = 50 µm. Sirius red staining (polarized light): yellow indicates type I collagen, and green indicates type III collagen. (F–H) Comprehensive evaluation of anti‐scar effects and promotion of wound healing in regenerated wounds, including (F) epidermal thickness, (G) collagen I/III ratio, and (H) collagen alignment index (n ≥ 3). Data are means ± SD. ^*^ *p* < 0.05, ^**^ *p* < 0.01, ^***^ *p* < 0.001, ns, *p* ≥ 0.05.

To determine the actual persistence time of nanoparticles in wound tissues and whether they retain targeting capability during the late stage of wound healing, ICP‐MS was performed to quantify the Ce and Cu contents in wound tissues at days 1, 3, 5, 7, and 10 after CAF@MN application. The results showed that the levels of both elements gradually decreased over time but remained clearly detectable at day 10. As shown in Figure S27A,B, the relative contents of Ce and Cu in wound tissues at days 1, 3, 5, 7, and 10 exhibited a similar decreasing trend compared with the initial amount of materials, indicating that Cu‐CeO_2_‐derived components could continuously remain in wound tissues after release. Furthermore, in vivo fluorescence imaging in small animals demonstrated that FITC‐labeled materials were detectable in the wound area at days 1, 3, 5, 7, and 10, with the relative fluorescence intensities shown in Figure S27C,D. Considering that the transition of fibroblasts into myofibroblasts begins around day 3 and increases rapidly during days 3–7, day 10 was selected as an extended time point after day 7 to evaluate whether material‐associated signals could persist into the early remodeling stage [52, 53]. Previous studies have also demonstrated that FITC covalently conjugated to peptides or hydrogel materials can remain detectable in vivo for more than 7 days [54, 55]. Collectively, the ICP‐MS and in vivo fluorescence imaging results demonstrate that CAF@MN‐derived components can persist until the middle‐to‐late stages of wound healing, thereby providing a temporal and material basis for FTP‐mediated targeting of FAP^+^ myofibroblasts and subsequent anti‐fibrotic effects.

Given that the nanoparticles had already been confirmed to exhibit good in vitro biosafety, further in vivo biosafety evaluation was conducted by systematically assessing body weight changes, blood biochemical parameters, oxidative stress‐related antioxidant indicators, and histological characteristics of major organs in the experimental mice [56]. The results showed that on postoperative days 1, 3, 7, and 14, compared with the Control group, no abnormalities were observed in body weight or in levels of alanine aminotransferase (ALT), urea (UREA), creatine kinase (CK), blood glucose (GLU), or triglycerides (TG) in the experimental groups (Figure S28n). Meanwhile, the levels of total superoxide dismutase (T‐SOD), reduced glutathione (GSH), catalase (CAT), and total antioxidant capacity (T‐AOC) in wound tissues were all increased, indicating enhanced local antioxidant defense capacity and effective alleviation of the oxidative microenvironment (Figure S29). This antioxidant effect not only reduced ROS‐mediated tissue damage and inflammatory responses but also provided a favorable microenvironment for re‐epithelialization, collagen remodeling, and scar inhibition. In addition, H&E staining‐based histological analysis of the heart, liver, spleen, lung, kidney, and brain tissues on postoperative day 14 revealed clearly identifiable typical structures such as splenic nodules, glomeruli, alveoli, and central veins, with no obvious inflammatory cell infiltration, tissue necrosis, or structural abnormalities observed in any group (Figure S30). Taken together, these in vivo results consistently demonstrate that CAF@MN significantly promotes wound healing in mice, markedly reduces hypertrophic scars, and that this nanomaterial system exhibits good in vivo biocompatibility and safety.

In addition, to verify the antibacterial performance of the nanoparticles in the in vivo environment, mouse wound infection models were established using MRSA and *E. coli*, respectively. Wound tissues were collected on postoperative day 3 for SEM observation and plate counting assays. The results showed that, in the Control group, typical rod‐shaped *E. coli* and spherical MRSA were clearly observed to grow together under SEM, indicating successful establishment of the infected wound model in mice (Figure S31). Among all treatment groups, the CAF@MN group exhibited the lowest colony numbers and the lowest relative bacterial viability, demonstrating the most effective in vivo antibacterial performance (Figure 6C and Figures S32–S33). It should be further clarified that the antibacterial effect observed in this study and the antioxidant effect observed in the animal wound model represent redox effects occurring at different levels and do not constitute a direct contradiction. In the antibacterial system, Cu‐CeO_2_ directly interacts with bacteria, where Cu‐related components can interact with sulfhydryl and amino groups on bacterial membrane surfaces, leading to membrane disruption and interference with the normal functions of key enzymes [47, 57]. Meanwhile, Cu‐mediated redox reactions can promote local ROS accumulation in bacteria and disrupt their redox homeostasis, with the Ce^3^^+^/Ce^4^^+^ cycling and surface oxygen vacancies potentially further contributing to this process [35, 58]. In contrast, the animal experiments reflect the overall redox state of intact wound tissues after treatment with the material. Cu‐CeO_2_ can scavenge excessive O_2_•^−^, •OH, and H_2_O_2_ through the reversible Ce^3^^+^/Ce^4^^+^ cycle and surface oxygen vacancies, while promoting H_2_O_2_ decomposition, thereby enhancing the levels of T‐SOD, GSH, CAT, and T‐AOC in wound tissues [57]. In addition, by reducing bacterial burden, the material can further decrease infection‐induced inflammatory responses and persistent endogenous ROS production [59]. Therefore, Cu‐CeO_2_ promotes localized oxidative damage at the direct bacteria–material interface to exert antibacterial effects, while simultaneously eliminating excessive ROS and restoring redox homeostasis in the overall wound tissue. These two effects are highly dependent on the target cells and microenvironmental conditions, rather than being mutually contradictory. Furthermore, considering that Cu can promote the generation and release of NO, which is beneficial for vascular formation and reconstruction during wound regeneration [37], regenerated skin tissues were collected on postoperative day 7 to measure NO levels. The results showed that the NO concentrations in regenerated skin tissues from the Cu‐containing C@MN, CA@MN, and CAF@MN groups were significantly higher than those in the Cu‐free Control and A@MN groups (Figure 6D), indicating that Cu effectively promotes NO synthesis and release in regenerated tissues, thereby creating a favorable microenvironment for angiogenesis.

To further evaluate wound healing in mice at the histological level, regenerated skin tissues from each group were harvested on day 14 after wound healing and subjected to H&E, Masson's trichrome, and Sirius red staining, followed by observation under conventional light microscopy and polarized light microscopy. H&E staining results showed that, compared with the Control group and other experimental groups, the CAF@MN group exhibited a more intact epidermal structure, a dermal morphology closer to that of normal skin, markedly reduced inflammatory cell infiltration, a thinner epidermis, and the presence of regenerated hair follicle‐like structures. Masson's trichrome and Sirius red staining (under normal light) revealed that collagen fibers in the Control group were thick, densely packed, and displayed a pronounced parallel orientation, consistent with typical histological features of fibrosis, whereas these features were alleviated to varying degrees in the experimental groups. This difference was more pronounced under polarized light, where the CAF@MN group showed an increased proportion of type III collagen (green), with relatively finer collagen fibers arranged in a looser, reticular pattern, consistent with anti‐fibrotic histological characteristics and favorable for scarless wound healing (Figure 6E). Meanwhile, quantitative analyses of epidermal thickness, the type I/type III collagen ratio, and the collagen fiber alignment index were further performed to comprehensively evaluate regenerated skin tissues [10]. The results showed that, compared with the other experimental groups, the CAF@MN group exhibited a thinner epidermis (28.94 µm, Figure 6F), a lower type I/type III collagen ratio (1.98, Figure 6G), and a collagen fiber arrangement pattern more similar to that of normal skin (0.34, Figure 6H). These findings indicate that CAF@MN can significantly promote skin tissue regeneration and collagen remodeling while effectively suppressing hypertrophic scars. resulting in regenerated tissue that more closely resembles normal skin in both structure and composition. In combination with the above results, this pro‐healing effect may be closely associated with its ability to promote NO release and its targeted regulation of macrophages and myofibroblasts.

### 2.7 CAF@MN Promotes Rabbit Whole‐Phase Skin Wound Healing and Tissue Regeneration and Inhibits Hypertrophic Scar

Unlike mouse skin wound healing, which mainly relies on wound‐edge contraction, rabbit ear skin wounds exhibit limited contraction, and their repair process depends more on re‐epithelialization as well as collagen deposition and remodeling. Therefore, this model has been widely used as a classical animal model for anti‐scar studies [60]. Based on the mouse experiments, a rabbit ear whole‐phase skin wound healing model was further introduced in this study, and the model construction process is shown in Figure S34. This model more closely mimics the human skin wound healing process and allows focused evaluation of the anti‐scar and regeneration‐promoting abilities of the materials. As shown in Figure 7A, the wound healing processes of rabbit ear whole‐phase skin wounds under different treatments were recorded on postoperative days 0, 5, 10, 20, and 30. In the Control and A@MN groups, wound closure was slow, and obvious scab residues and irregular tissue hyperplasia were observed during healing, indicating a tendency toward hypertrophic scar. In contrast, the C@MN and CA@MN groups showed accelerated wound repair; however, varying degrees of tissue roughness and uneven coloration were still observed at the middle and late stages. The CAF@MN group exhibited faster wound closure at all time points, with the wound surface gradually becoming smooth and uniform in color. By postoperative day 30, wounds in the CAF@MN group were nearly completely healed with the lowest degree of hypertrophic scar, which was more evident in lateral observations (Figure S35). The regenerated wound tissue in the CAF@MN group more closely resembled normal skin in gross appearance, demonstrating significantly superior skin regeneration and anti‐scar effects compared with the other treatment groups.

**FIGURE 7:**
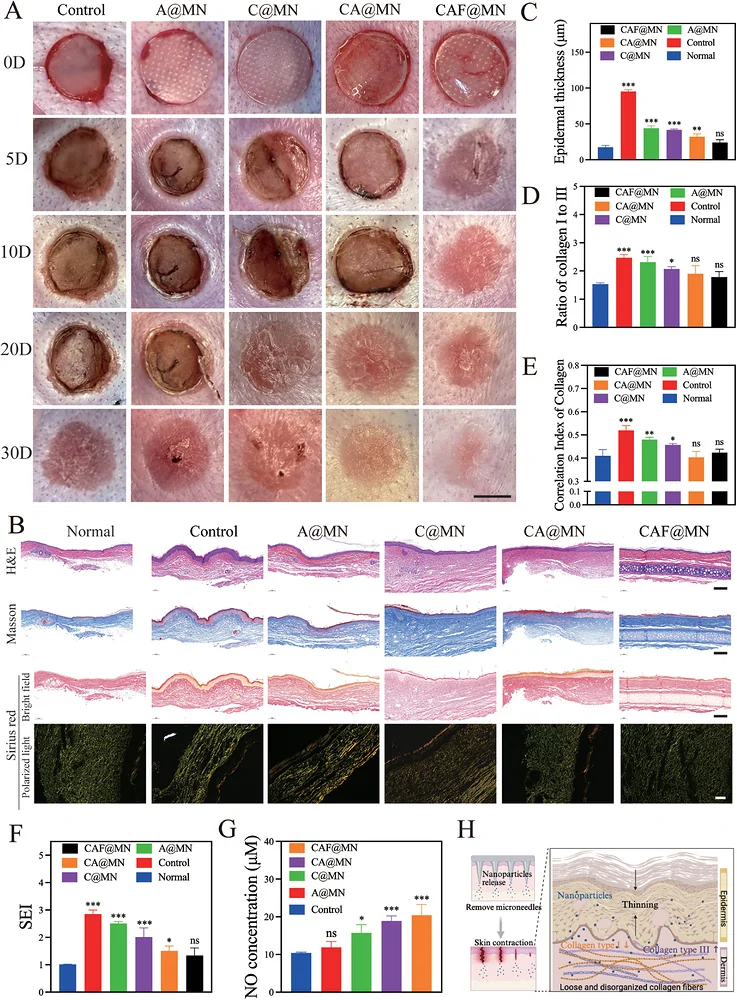
(A) Photographs of wounds in rabbits under different treatment conditions at different time points. Scale bar = 5 mm. (B) Representative images of H&E staining, Masson's trichrome staining, and Sirius red staining (bright‐field and polarized light) of wound tissues on day 30. Scale bar = 50 µm. Sirius red staining (polarized light): yellow indicates type I collagen, and green indicates type III collagen. (C–E) Comprehensive evaluation of anti‐scar effects and promotion of wound healing in regenerated wounds, including (C) epidermal thickness, (D) collagen I/III ratio, and (E) collagen alignment index (n ≥ 3). (F) Comparison of the Scar Elevation Index (SEI) in wound tissues among different treatment groups (n = 3). (G) Comparison of NO levels in wound tissues of rabbits from different treatment groups (n = 3). (H) Schematic illustration showing that CAF@MN acts on wound tissues to release nanoparticles, thereby promoting skin tissue regeneration and regulating collagen fiber remodeling to achieve wound repair. Data are means ± SD. ^*^ *p* < 0.05, ^**^ *p* < 0.01, ^***^ *p* < 0.001, ns, *p* ≥ 0.05.

To further evaluate wound healing of rabbit ear skin at the histological level, regenerated skin tissues from rabbit ears in each group were harvested on day 30 after wound healing and subjected to H&E, Masson's trichrome, and Sirius red staining, followed by observation under conventional light microscopy and polarized light microscopy. H&E staining results showed that epidermal thickening and disorganized tissue architecture were observed in the Control and A@MN groups, whereas the CAF@MN group exhibited a continuous and intact epidermis with thin and uniform thickness, a clearly defined dermal structure, and an overall morphology most closely resembling normal skin. Masson's trichrome staining indicated enhanced collagen staining in the Control group, suggesting a pronounced fibrotic tendency; in contrast, the CAF@MN group displayed moderate collagen deposition with a uniform distribution and no obvious excessive accumulation. Sirius red staining under both normal and polarized light further revealed that the Control group was dominated by thick, densely packed, and parallel‐aligned collagen fibers, whereas the experimental groups showed varying degrees of improvement. Among them, the CAF@MN group exhibited the most pronounced improvement, characterized by predominantly fine, reticular collagen fibers and an increased proportion of type III collagen (green), presenting collagen remodeling features more similar to normal skin [61] (Figure 7B).

Quantitative analyses of epidermal thickness, the type I/type III collagen ratio, and the collagen fiber alignment index were further performed in regenerated rabbit ear skin tissues. The results showed that, compared with the Control group and other treatment groups, the CAF@MN group exhibited a significantly reduced epidermal thickness (24.07 µm, Figure 7C), a lower type I/type III collagen ratio (1.79, Figure 7D), and a collagen fiber alignment index (0.42, Figure 7E) closer to that of normal skin, indicating a marked improvement in collagen remodeling quality and effective inhibition of hypertrophic scars. The Scar Elevation Index (SEI) objectively reflects the effects of materials on scar suppression and skin regeneration quality and is a commonly used evaluation parameter in wound repair research [62]. As shown in Figure 7F, compared with the Normal group, the Control group exhibited a significantly elevated SEI in regenerated rabbit ear wounds, indicating disordered collagen fiber arrangement and an increased degree of scarring. The SEI values were reduced in the experimental groups, with the CAF@MN group showing a markedly decreased SEI approaching that of the Normal group, demonstrating that CAF@MN effectively promotes orderly collagen reconstruction and suppresses hypertrophic scar. In addition, on postoperative day 14 of rabbit ear wound healing, NO regeneration effects similar to those observed in mouse wounds were also detected, with significantly increased NO concentrations in regenerated tissues of the CAF@MN group, suggesting that these treatments effectively enhance local NO production and are conducive to promoting vascular regeneration and repair in wounds [37] (Figure 7G).

Taken together, these results demonstrate that CAF@MN significantly accelerates wound closure in the rabbit ear whole‐phase skin wound healing model and exhibits a smoother, more uniform appearance that closely resembles normal skin during the late stage of healing. At the microscopic level, CAF@MN effectively suppresses excessive epidermal thickening and abnormal collagen deposition, promotes an increased proportion of type III collagen, and drives collagen fibers to remodel from thick, parallel arrangements into finer, reticular structures, thereby markedly reducing the SEI and improving the orderliness of collagen organization; meanwhile, NO levels in regenerated wound tissues are elevated. Collectively, these changes indicate that CAF@MN is conducive to high‐quality skin regeneration and exerts a pronounced anti‐scar reparative effect (Figure 7H).

### 2.8 ScRNA‐seq Analysis of CAF@MN‐Promoted Wound Healing

The above studies systematically demonstrated, through in vitro and in vivo experiments, that these nanoparticles exhibit favorable effects in promoting wound healing and inhibiting hypertrophic scars. Furthermore, on postoperative day 14, regenerated wound tissues from mice were collected, and three samples each from the Control group and the CAF@MN group were randomly selected for scRNA‐seq analysis to dissect, at single‐cell resolution, the compositional characteristics of key cellular subpopulations and their molecular regulatory mechanisms during wound healing [63]. A total of six samples were included in this analysis, yielding 76 839 valid single cells. The average median number of genes detected per cell was 2422, and a total of 2.24 × 10^9^ reads were obtained across all samples.

Single‐cell Uniform Manifold Approximation and Projection (UMAP) clustering analysis showed [64] that cells in regenerated mouse skin tissues could be clearly classified into major types, including T cells, B cells, fibroblasts (fib), macrophages (Mac), endothelial cells (Endo), myofibroblasts (MyoFib), keratinocytes (Kera), Schwann cells (Sch), and smooth muscle cells (SM). Each cell subpopulation exhibited good separation and well‐defined boundaries in low‐dimensional space (Figure 8A). The Control group and the CAF@MN group showed similar overall clustering architectures; however, obvious differences were observed in the distribution proportions and cellular states of specific cell populations, suggesting that CAF@MN treatment markedly influences immune regulation‐ and matrix remodeling‐related cellular behaviors in regenerated skin tissues (Figure S36). Gene expression bubble plots further demonstrated highly cell type‐specific marker gene expression patterns, with distinct molecular characteristics clearly distinguishing different cell populations (Figure 8B). In terms of cellular composition, compared with the Control group (inner circle), the experimental group (outer circle) exhibited increased proportions of immune cells in regenerated skin tissues, including T cells (27.3%→28.5%), B cells (14.6%→18.7%), and macrophages (8.6%→14.7%), whereas the proportions of fibroblasts (16.3%→11.7%) and myofibroblasts (3.4%→3.2%) were correspondingly reduced. These results indicate that CAF@MN treatment can effectively remodel the wound immune microenvironment and, to some extent, suppress the abnormal transition of fibroblasts to myofibroblasts, thereby facilitating the orderly progression of tissue repair [65] (Figure 8C and Figure S37).

**FIGURE 8:**
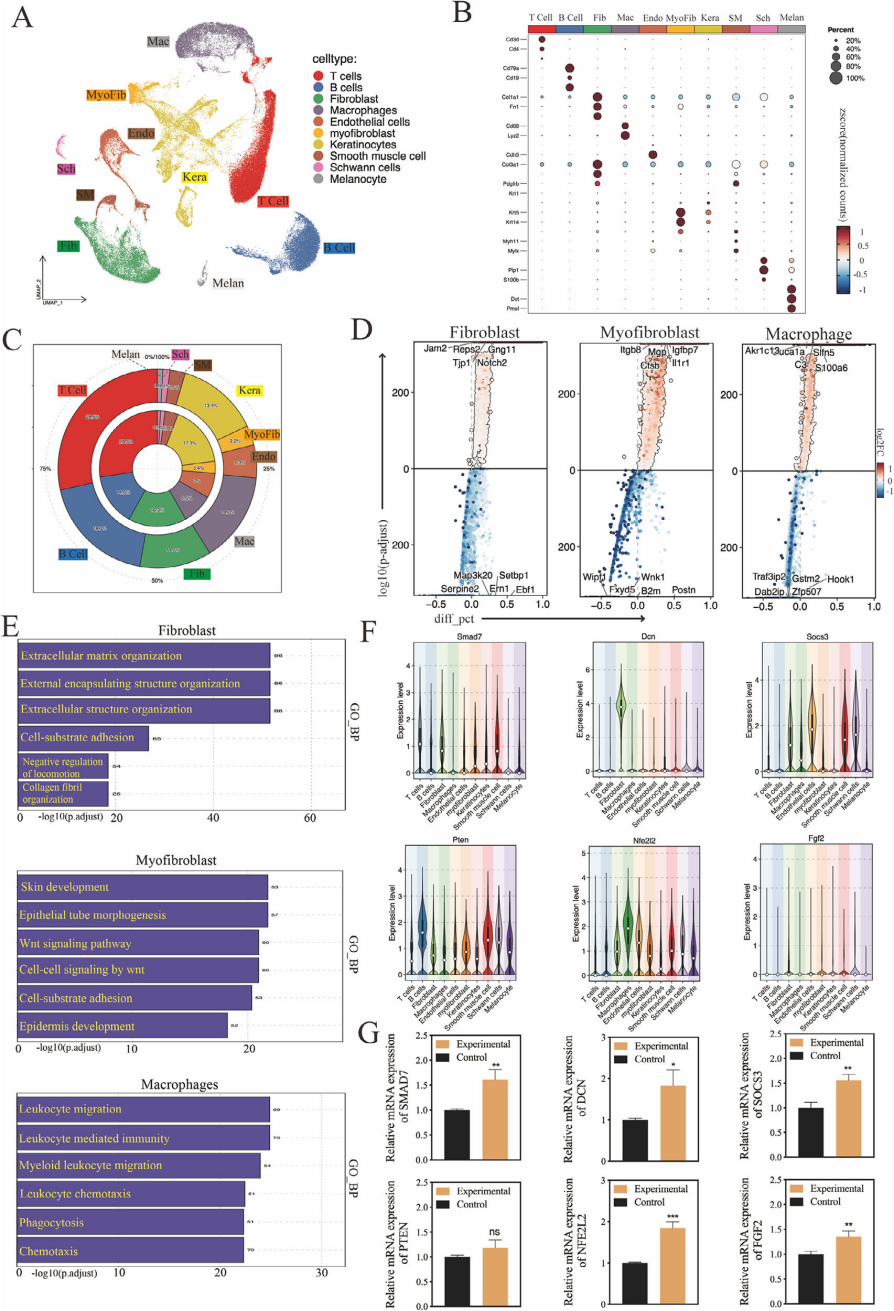
(A) Uniform Manifold Approximation and Projection (UMAP) visualization illustrates the distribution of different cell types in the single‐cell transcriptomic dataset (Mac, macrophages; MyoFib, myofibroblasts; Endo, endothelial cells; Kera, keratinocytes; Sch, Schwann cells; SM, smooth muscle cells; Fib, fibroblasts; Melan, melanocytes). (B) Dot plot showing marker gene expression across distinct cell types. (C) Circular plot showing the cellular composition across two experimental groups. (D) Volcano scatter plots of differentially expressed genes in fibroblasts, myofibroblasts, and macrophages. (E) Gene Ontology—biological process (GO‐BP) biological process enrichment analysis of fibroblasts, myofibroblasts, and macrophages. (F) Violin plots showing the expression levels of the key genes Smad7, Dcn, Socs3, Pten, Nfe2l2, and Fgf2 across different cell types. (G) RT‐qPCR validation of the relative mRNA expression levels of key genes (n = 3). Data are means ± SD. ^*^ *p* < 0.05, ^**^ *p* < 0.01, ^***^ *p* < 0.001, ns, *p* ≥ 0.05.

Differentially expressed genes (DEGs) analysis focusing on fibroblasts, myofibroblasts, and macrophages—the key cell types of interest in this study—revealed that different cell types exhibited cell type‐specific transcriptional response patterns to CAF@MN treatment and cooperatively participated in key biological processes, including regulation of inflammatory responses, matrix remodeling, and tissue regeneration (Figure 8D and Figure S38). Gene Ontology‐Biological Process (GO‐BP) enrichment analysis showed that fibroblasts were mainly enriched in biological processes related to extracellular matrix (ECM) organization, collagen structure formation, and remodeling; myofibroblasts were primarily associated with collagen remodeling and regulation of scar formation; whereas macrophages were significantly enriched in processes such as leukocyte migration, chemotaxis, phagocytosis, and immune responses. These results indicate that CAF@MN promotes skin regeneration by coordinately regulating immune responses and ECM remodeling [66] (Figure 8E and Figure S39).

In addition, genes closely associated with tissue repair and anti‐fibrotic processes, including Smad7, Dcn, Socs3, Pten, Nfe2l2, and Fgf2, were systematically analyzed across nine cell types. The results showed that, compared with other cell types, these genes exhibited more pronounced differential expression patterns in fibroblasts, myofibroblasts, and macrophages involved in wound repair (Figure 8F). Moreover, box plot analysis revealed obvious differential expression of these genes in the CAF@MN group compared with the Control group (Figure S40). Furthermore, HDFs treated with DMEM and CM were used as the control and experimental groups, respectively, and RT‐qPCR analysis of the above genes showed expression trends largely consistent with the scRNA‐seq results, indicating that CAF@MN can globally regulate multiple key genes closely related to wound healing (Figure 8G). Notably, PTEN exerts an inhibitory effect on cell proliferation and migration during the early stage of wound healing, whereas during the middle and late stages it can limit excessive activation of myofibroblasts and thereby exert anti‐fibrotic effects [67, 68]. Therefore, although upregulation of PTEN expression was observed in both scRNA‐seq and RT‐qPCR analyses, the difference did not reach statistical significance, which indirectly reflects the stage‐dependent and fine‐tuned regulatory characteristics of CAF@MN during the promotion of rapid and scarless wound healing.

Gene Set Enrichment Analysis (GSEA) was used to evaluate whether gene sets are globally enriched under different conditions, thereby revealing pathway alterations and underlying biological mechanisms [69]. In fibroblasts and myofibroblasts from the CAF@MN group, pathways related to ECM organization, cell adhesion, and tissue remodeling were significantly enriched, whereas pathways associated with inflammation and stress responses were suppressed. Meanwhile, in macrophages, pathways related to immune responses, leukocyte‐mediated immunity, and phagocytosis were markedly enhanced (Figure 9A). T cells and B cells also played important synergistic roles in immune regulation (Figure S41). This differential regulation indicates that the targeting capability of the nanoparticles enables selective and precise actions on myofibroblasts and macrophages, driving them to exert distinct biological functions, and suggests that CAF@MN promotes high‐quality wound repair through coordinated regulation of the immune microenvironment and matrix remodeling.

**FIGURE 9:**
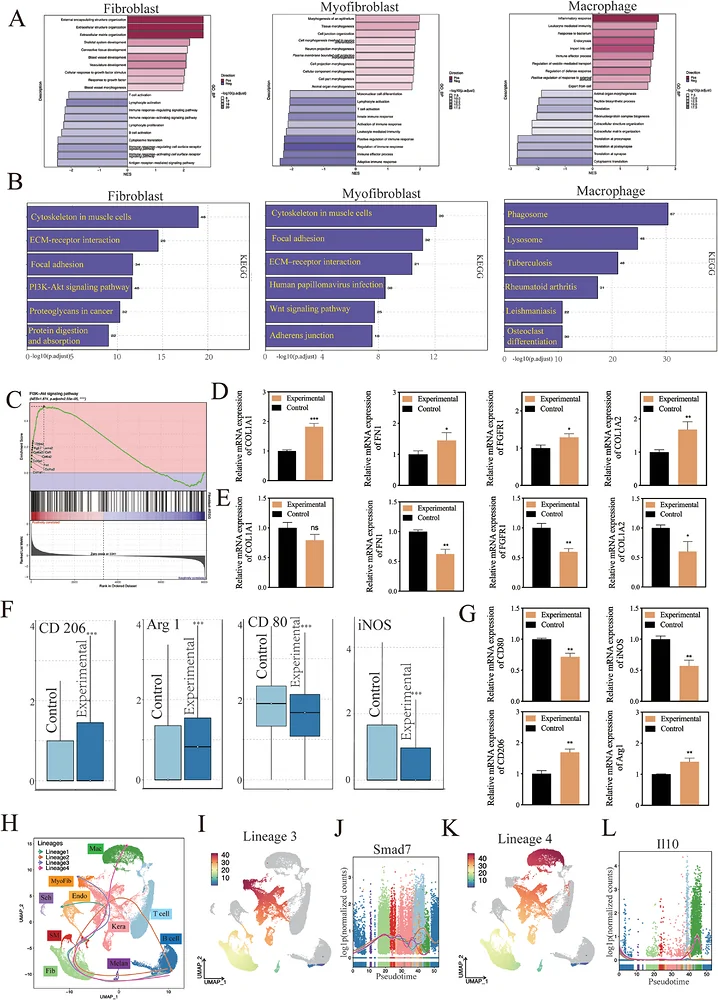
(A) GO‐BP enrichment analysis based on Gene Set Enrichment Analysis (GSEA) of fibroblasts, myofibroblasts, and macrophages. (B) Kyoto Encyclopedia of Genes and Genomes (KEGG) pathway enrichment analysis using GSEA of fibroblasts, myofibroblasts, and macrophages. (C) GSEA enrichment plot of the PI3K‐AKT signaling pathway in fibroblasts. (D) RT‐qPCR analysis of the relative mRNA expression levels of different genes in HDFs (n = 3). (E) RT‐qPCR analysis of the relative mRNA expression levels of different genes in myofibroblasts (n = 3). (F) scRNA‐seq‐based analysis of M1/M2 polarization‐related gene expression in macrophages. (G) RT‐qPCR validation of the relative mRNA expression levels of macrophage M1/M2 polarization‐related genes (n = 3). (H) Single‐cell trajectory analysis reveals lineage relationships and state transitions among distinct cell types. (I) Pseudotime distribution of lineage 3 in UMAP space. (J) Dynamic expression pattern of Smad7 along pseudotime. (K) Pseudotime distribution of lineage 4 in UMAP space. (L) Dynamic expression pattern of Il10 along pseudotime. Data are means ± SD. ^*^ *p* < 0.05, ^**^ *p* < 0.01, ^***^ *p* < 0.001, ns, *p* ≥ 0.05.

Further gene functional module analysis at the subcluster level revealed that fibroblasts and myofibroblasts were mainly involved in ECM remodeling, cell migration, and signal transduction processes, whereas macrophages were significantly enriched in pathways related to immune responses, inflammatory regulation, and phagocytosis. Overall, distinct cellular subpopulations exhibited clear functional specialization and cooperative interactions during wound repair, jointly driving tissue remodeling and optimization of the immune microenvironment (Figure S42). The Kyoto Encyclopedia of Genes and Genomes (KEGG) is commonly used for gene function annotation and pathway analysis [70]. KEGG analysis showed that fibroblasts were mainly enriched in pathways associated with cytoskeletal remodeling, adherens junctions, and ECM‐receptor interaction, whereas pathways related to contraction, adhesion, and ECM interactions were globally suppressed in myofibroblasts, indicating that their activation status was effectively restrained [5] (Figure 9B). Notably, the PI3K‐AKT signaling pathway was significantly enriched in fibroblasts but did not show significant activation in myofibroblasts, with its associated genes generally exhibiting a low expression trend, suggesting restricted pathway activity that may help inhibit excessive myofibroblast activation and fibrotic progression (Figure 9C and Figure S43). To further validate these findings, HDFs and myofibroblasts were treated with DMEM and CM as the control and experimental groups, respectively, and key genes within the PI3K‐AKT pathway were selected for reverse validation in HDFs. RT‐qPCR results showed that the expression trends of COL1A1, FN1, FGFR1, and COL1A2 were consistent with the scRNA‐seq‐based PI3K‐AKT pathway analysis (Figure 9D,E). Collectively, these results demonstrate that CAF@MN can promote fibroblast reparative functions while suppressing excessive myofibroblast activation‐associated hypertrophic scar through differential regulation of the PI3K‐AKT pathway, thereby playing an important role in promoting scarless wound healing [71]. To further validate the changes in the PI3K‐AKT signaling pathway at the protein level, Western blot analysis was performed on HDFs treated with Control or CAF@MN. The results showed that there were no significant differences in the expression levels of total PI3K and AKT between the two groups, whereas the levels of p‐PI3K and p‐AKT were significantly increased in the CAF@MN‐treated group (Figure S44A,B). These results were consistent with the trends observed in the scRNA‐seq and RT‐qPCR analyses, further supporting the involvement of the PI3K‐AKT signaling pathway in CAF@MN‐mediated enhancement of fibroblast repair functions.

In addition, the scRNA‐seq results of this study systematically revealed the mechanism by which CAF@MN regulates inflammatory responses during wound healing at the single‐cell level. The results showed that the M1 marker gene CD80 was generally expressed at low levels across various cell types, whereas the M2 marker gene CD206 was highly expressed in macrophages, indicating that the wound microenvironment in the experimental group was dominated by a reparative macrophage phenotype (Figure S45). Further scRNA‐seq analysis demonstrated that, compared with the Control group, macrophages in the CAF@MN group exhibited upregulated expression of the M2 marker genes CD206 and Arg1, while the expression of the M1 marker genes CD80 and iNOS was downregulated, indicating a phenotypic shift of macrophages toward M2 polarization (Figure 9F). To experimentally validate the scRNA‐seq findings, RAW 264.7 macrophages treated with DMEM and CM derived from the CAF@MN group were used as the control and experimental groups, respectively, for RT‐qPCR analysis. The results showed that the expression changes of the relevant genes were consistent with the scRNA‐seq analysis (Figure 9G). Collectively, these findings further confirm, at the single‐cell level, the conclusions drawn from previous experiments, namely that CAF@MN can target and regulate macrophages and promote their polarization toward the M2 phenotype, thereby remodeling the immune microenvironment, enhancing anti‐inflammatory responses, and ultimately providing favorable conditions for wound healing.

Finally, to elucidate the dynamic evolution of cellular states and key regulatory mechanisms during wound healing, pseudotime analysis was further performed [63]. As shown in Figure 9H, different cell types exhibited continuous state transitions along four lineage trajectories in UMAP space. Lineage 1 mainly evolved toward endothelial cells and Schwann cells, whereas Lineage 2 predominantly differentiated toward immune cells such as macrophages, T cells and B cells, indicating that vascular/neural remodeling and immune regulation represent two major concurrent trajectories during wound repair (Figure S46). Among these, cells in Lineage 3 progressed along a single continuous trajectory from early to late states, primarily reflecting the transition from fibroblasts to myofibroblasts (Figure 9I). Given that Smad7 participates in anti‐fibrotic regulation through modulation of the TGF‐β/SMAD signaling pathway, the dynamic expression of Smad7 along Lineage 3 was analyzed. During wound healing, elevated Smad7 expression in myofibroblasts exerts anti‐fibrotic effects. Notably, at the late stage of wound healing, Smad7 is highly expressed in T cells and macrophages, which is beneficial for alleviating inflammatory responses and ROS‐induced damage. This expression pattern is highly consistent with the stage‐dependent regulatory effects exhibited by the nanoparticles during the wound healing process. To further validate SMAD7‐related anti‐fibrotic signaling, the expression levels of SMAD7 and SMAD2/3‐related proteins were examined in HDFs treated with conditioned media from the Control and CAF@MN groups. Western blot analysis showed that SMAD7 protein expression was significantly increased in the CAF@MN group, whereas the expression levels of total SMAD2 and SMAD3 remained unchanged. Meanwhile, the p‐SMAD2/SMAD2 and p‐SMAD3/SMAD3 levels were markedly decreased (Figure S44C,D). These results suggest that CAF@MN may upregulate SMAD7 and inhibit SMAD2/3 phosphorylation, thereby limiting excessive activation of the canonical TGF‐β/SMAD signaling pathway and attenuating the pro‐fibrotic phenotype of myofibroblasts. Pseudotime analysis of Lineage 4 mainly corresponded to the evolutionary trajectory of macrophages, in which cells gradually transitioned from low to high pseudotime states along a continuous trajectory in UMAP space, forming a clear temporal gradient (Figure 9K). As an important marker gene of macrophage M2 polarization, IL10 exhibited high expression within the macrophage population in the Lineage 4 pseudotime analysis (Figure 9L), indicating markedly enhanced M2 polarization of macrophages. This further supports the mechanism by which the nanoparticles suppress wound inflammation by promoting macrophage polarization toward an anti‐inflammatory phenotype. In addition, multiple genes displayed stage‐specific expression changes along different trajectories in the pseudotime analysis, including anti‐fibrotic genes such as Pten and anti‐inflammatory and immunoregulatory genes such as Pparg [67, 72]. These findings indicate that the wound healing process gradually transitions from inflammation resolution to tissue repair and matrix remodeling, and reveal the temporal characteristics of coordinated regulation among multiple cell types during the later stages of healing (Figure S47).

Taken together, scRNA‐seq analysis at single‐cell resolution systematically elucidated the CAF@MN‐mediated regulation of wound healing, including changes in cellular composition, functional states, and underlying molecular mechanisms. At the single‐cell level, these findings further validate the aforementioned experimental results, demonstrating that CAF@MN promotes polarization of immune cells—particularly macrophages—toward the M2 anti‐inflammatory phenotype, thereby suppressing inflammatory responses. Meanwhile, CAF@MN exerts stage‐dependent and differential regulation of fibroblasts and myofibroblasts, enhancing fibroblast reparative activity while restraining excessive activation and fibrotic progression of myofibroblasts. Pseudotime analysis further revealed the dynamic temporal evolution of key cells and genes during wound healing, providing robust single‐cell‐level evidence for the stage‐specific and targeted regulatory effects of the nanoparticles.

## 3 Conclusion

This study addresses the challenges of delayed healing, persistent inflammation, and hypertrophic scar that remain prevalent in the field of skin wound repair by designing and constructing a dual‐targeting biomimetic nanozyme hybrid system, Cu‐CeO_2_@ABs‐FTP, which was further integrated with HAMA hydrogel to develop the CAF@MN therapeutic system. The design strategy is as follows: (1) first, Cu was introduced onto CeO_2_ nanoparticles to synthesize Cu‐CeO_2_ nanoparticles with anti‐inflammatory, antibacterial, antioxidative, and pro‐angiogenic functions; (2) second, the surface of Cu‐CeO_2_ nanoparticles was coated with ABs membrane derived from Jurkat T cells, endowing the nanoparticles with the ability to target macrophages in inflammatory regions and to promote macrophage polarization toward the M2 phenotype; (3) subsequently, based on the N_3_ assembled on the ABs membrane surface and the DBCO modification at one end of FTP, FTP was conjugated onto the nanoparticle surface via a click chemistry reaction to generate Cu‐CeO_2_@ABs‐FTP, thereby conferring targeting capability toward myofibroblasts; and (4) finally, taking advantage of the ability of microneedles to enhance localized deep delivery and utilization efficiency, Cu‐CeO_2_@ABs‐FTP was combined with HAMA hydrogel to construct the CAF@MN therapeutic system.

This study demonstrated the dual‐targeting capability and multiple biological effects of the CAF@MN therapeutic system through in vitro experiments. In addition, mouse and rabbit whole‐phase skin wound healing models were established to validate, at the in vivo level, the integrated therapeutic effects of CAF@MN in promoting tissue repair, controlling inflammation, and suppressing hypertrophic scars. Finally, scRNA‐seq analysis of mouse wound tissues was performed to further confirm, at single‐cell resolution, that the CAF@MN therapeutic system activates fibroblasts, suppresses myofibroblasts, and promotes macrophage polarization toward the M2 phenotype. Moreover, the underlying biological mechanisms were elucidated, revealing that the nanoparticles exert these effects through regulation of the PI3K‐AKT pathway as well as genes such as Smad7 and Pten. Pseudotime analysis was further applied to investigate the dynamic temporal evolution of these cells and genes during wound healing, providing mechanistic insights into how the material functions at the single‐cell level.

In summary, this dual‐targeting multifunctional therapeutic system functionally surpasses conventional hydrogel wound dressings and provides a promising therapeutic strategy for enhancing wound repair while suppressing inflammatory responses and hypertrophic scars. However, it should be noted that previous studies have reported that, under certain pathological conditions such as keloids, a subset of fibroblasts that are functionally hyperactive but have not yet differentiated into myofibroblasts can also exhibit high FAP expression. These cells may competitively bind nanoparticles with FAP‐high myofibroblasts. Therefore, under specific disease conditions, how to accurately define and distinguish these two cell populations, as well as how to further enhance the competitive binding of nanoparticles to myofibroblasts, remains a challenge. Nevertheless, as an interdisciplinary strategy integrating tissue engineering and biomedical engineering, the design concept of this dual‐targeting biomimetic nanozyme hybrid system still offers an innovative biomaterial candidate for addressing the multiple challenges encountered during wound healing.

## 4 Methods

### 4.1 Materials

The Annexin V‐FITC/PI apoptosis detection kit, dihydroethidium, and Cell Counting Kit‐8 (CCK‐8) were purchased from Shanghai Beyotime Biotechnology Co., Ltd. Tetraethyl orthosilicate (TEOS), ethanol, ammonia solution, cerium acetylacetonate, copper chloride, L‐ascorbic acid, dialysis tubing with a molecular weight cutoff of 10 kDa, Cy5.5 NHS ester, and FITC were obtained from Aladdin Reagent Co., Ltd. DMEM and RPMI‐1640 media, fetal bovine serum (FBS), penicillin‐streptomycin solution, and 0.25% trypsin were purchased from Gibco. PBS buffer was obtained from HyClone. Antibodies, including FITC‐anti‐CD206 and APC‐anti‐F4/80 were purchased from BioLegend (USA). Basic fibroblast growth factor (bFGF) was purchased from Shanghai PrimeGene Bio‐Tech Co., Ltd. (China).

### 4.2 Characterization

The hydrodynamic size and zeta potential of the nanoparticles were measured at 298.0 K using a nanoparticle size and zeta potential analyzer (Zetasizer Nano ZS90, Malvern, UK). The surface chemical composition of the nanoparticles was analyzed by X‐ray photoelectron spectroscopy (XPS, K‐Alpha+, Thermo Fisher Scientific, USA). The crystalline structure and phase composition of the nanoparticles were characterized using an X‐ray diffractometer (XRD, Ultima IV). The micro‐morphology of the nanoparticles was observed by transmission electron microscopy (TEM, JEM‐2010), while the morphology of the microneedles and bacteria was characterized by scanning electron microscopy (SEM, JSM‐7800F, Japan). In addition, HAMA hydrogel microneedles were labeled with FITC fluorescent dye and observed using a confocal laser scanning microscope (STELLARIS 5, Leica Microsystems, Germany).

### 4.3 Synthesis of Nanoparticles

SiO_2_ nanospheres were first synthesized using the Stöber method. Briefly, 50 mL ethanol, 2 mL deionized water, and 2 mL ammonia solution were mixed thoroughly, after which 3 mL tetraethyl orthosilicate (TEOS) was added under continuous stirring and allowed to react for 4–6 h. After completion, the products were collected by centrifugation and washed three times each with ethanol and deionized water to obtain SiO_2_ nanospheres with an average diameter of approximately 100 nm, followed by characterization of their particle size and zeta potential. Subsequently, CeO_2_ shells were constructed using the SiO_2_ nanospheres as templates. Specifically, 35 mg of SiO_2_ was dispersed in 60 mL deionized water and subjected to bath sonication and mechanical stirring for 30 min, followed by the addition of cerium acetylacetonate (150 mg) and urea (171.4 mg). After stirring for an additional 1 h, the mixture was transferred to an autoclave and reacted hydrothermally at 160°C for 6 h to obtain hollow CeO_2_ (HCeO_2_) nanoparticles. Then, 40 mg of HCeO_2_ was dispersed in 20 mL of deionized water, and 200 mg of poly (allylamine hydrochloride) (PAH) was added. After stirring for 2 h, positively charged HCeO_2_ nanoparticles were collected by centrifugation.

To prepare copper nanoparticles, 10 mmol copper chloride was dispersed in 50 mL deionized water and magnetically stirred at 80°C for 10 min. Subsequently, 50 mL of 400 mM L‐ascorbic acid solution was added dropwise, and the reaction was maintained at 80°C for 16 h under stirring. After completion, the mixture was centrifuged (6577 × g, 15 min) to remove large aggregates. The supernatant was then transferred into dialysis tubing with a molecular weight cutoff of 10 kDa and dialyzed against deionized water for 48 h to remove unreacted small molecules. Next, 40 mg of positively charged HCeO_2_ was added to the obtained copper nanoparticle dispersion and stirred for 12 h. The resulting product was washed three times by centrifugation with deionized water to construct Cu‐CeO_2_ composite nanoparticles (Figure S3).

To obtain Cu‐CeO_2_@ABs, pre‐isolated and purified Jurkat T cell‐derived ABs were mixed with Cu‐CeO_2_ nanoparticles at a predefined mass ratio of 1:1 and incubated in an ice bath to facilitate initial adsorption. The mixture was then repeatedly extruded through polycarbonate membranes with a pore size of 200 nm, driving uniform coating of the ABs membrane onto the Cu‐CeO_2_ surface under mechanical shear to construct Cu‐CeO_2_@ABs. TEM imaging of Cu‐CeO_2_@ABs nanoparticles confirmed successful membrane coating on the nanoparticle surface. Subsequently, N_3_‐modified Cu‐CeO_2_@ABs were incubated with DBCO‐FTP (Cys‐His‐Gly‐Pro‐Arg, FITC‐labeled) under mild conditions, enabling efficient covalent conjugation via click chemistry. After the reaction, the product was collected by centrifugation and washing to obtain the Cu‐CeO_2_@ABs‐FTP nanosystem for subsequent experiments.

### 4.4 Histology and Immunofluorescence

After fixation in 4% paraformaldehyde, tissue samples were subjected to routine dehydration, clearing, and paraffin embedding, and serial tissue sections with a thickness of approximately 4–5 µm were prepared for histological analysis. Following deparaffinization in xylene and rehydration through a graded ethanol series, the sections were stained with hematoxylin and eosin (H&E) to evaluate epidermal re‐epithelialization, granulation tissue formation, and inflammatory cell infiltration. Masson's trichrome staining was performed to assess collagen deposition and dermal structural reconstruction. Sirius red staining was conducted and imaged under both bright‐field and polarized light conditions to analyze the distribution characteristics and alignment of different collagen fiber types. All tissue sections were imaged using an optical microscope, and relevant histological parameters, including epidermal thickness, granulation tissue thickness, and collagen distribution, were subjected to semi‐quantitative or quantitative analysis using ImageJ software, thereby enabling a comprehensive evaluation of skin wound repair and hypertrophic scar in mice and rabbits.

### 4.5 Mechanical Properties Testing

The mechanical properties of HAMA hydrogel microneedles with different formulations were evaluated by compression testing using a universal testing machine (Instron 5969). During testing, the microneedle arrays were compressed vertically, and force‐displacement response curves were recorded to assess the mechanical stability of the microneedle system. The testing setup and representative photographs of the experimental procedure are shown in Figure S19.

### 4.6 Cell Proliferation and Migration Assays

Cell viability of human dermal fibroblasts (HDFs) was evaluated using the CCK‐8 cell counting kit (Beyotime, China). Nanoparticles were prepared as conditioned media (CM) at different concentrations (0, 6.25, 12.5, 25, 50, 100, 200, and 400 µg mL^−1^) and used to treat HDFs. At the indicated time points, 10 µL of CCK‐8 reagent was added to each well of a 96‐well plate, followed by incubation at 37°C for 2 h. The absorbance was then measured at 450 nm using a microplate reader (BioTek Epoch, USA).

Cell migration was assessed by a scratch assay. HDFs were seeded into 24‐well plates and cultured until approximately 80% confluence. A straight scratch was created vertically across the cell monolayer using a 200 µL pipette tip, followed by gentle washing with PBS 2–3 times to remove detached cells. Cells were then cultured in different conditioned media. Images were acquired at 24 h using a confocal laser scanning microscope (STELLARIS 5, Leica Microsystems, Germany), and the scratch area was analyzed using ImageJ software. The cell migration rate was calculated as the ratio of the healed scratch area to the initial scratch area.

### 4.7 Flow Cytometry Analysis of Macrophages

RAW 264.7 cells (IM‐MOCELL, Xiamen, China) were collected after the indicated treatments and washed with PBS. The cells were then incubated with anti‐F4/80 antibody (BioLegend, USA) and anti‐CD206 antibody (BioLegend, USA) for 0.5 h in the dark. After staining, the cells were collected by centrifugation and washed with PBS. Finally, samples were analyzed by flow cytometry to evaluate the proportions of different macrophage subpopulations. All data were acquired using a CytoFLEX flow cytometer and analyzed with FlowJo 10 software.

### 4.8 Quantitative Gene Expression Analysis

Total cellular RNA was extracted using TRIzol reagent (Thermo Fisher Scientific, Dreieich, Germany). RNA concentration and purity were assessed by measuring the absorbance at 260/280 nm using a NanoDrop spectrophotometer (Thermo Fisher Scientific, Dreieich, Germany). Real‐time quantitative PCR was performed using the Hifair III One Step RT‐qPCR SYBR Green Kit (Yeasen, China), with total RNA as the template, allowing reverse transcription and PCR amplification to be carried out in the same reaction system. The reaction conditions were set as follows: reverse transcription at 42°C for 15 min, initial denaturation at 95°C for 5 min, followed by 40 amplification cycles (95°C for denaturation and 60°C for annealing/extension), during which fluorescence signals were collected in real time. Gene expression levels were normalized to internal reference genes such as GAPDH/β‐actin. The primer sequences are listed in Table S1.

### 4.9 Preparation of Unmodified Hama Hydrogel

Fifty milligrams of lithium phenyl‐2,4,6‐trimethylbenzoylphosphinate (LAP) was added to 20 mL PBS buffer as a photoinitiator and heated at 50°C until fully dissolved, followed by storage under dark conditions at low temperature for later use. Subsequently, 0.1 g of HAMA powder was dissolved in 2 mL of the pre‐prepared photoinitiator solution and heated with stirring at 60°C to obtain a homogeneous 5% (w/v) HAMA hydrogel precursor solution. The resulting precursor solution was stored at 4°C prior to use.

### 4.10 Preparation of Nanoparticle‐Modified HAMA Hydrogel Dressings

Taking C@MN as an example, the pre‐prepared HAMA hydrogel precursor system was placed at room temperature, and an appropriate amount of Cu‐CeO_2_ nanoparticles was added. The mixture was gently vortexed or slowly stirred to ensure homogeneous dispersion of the nanoparticles, with the final nanoparticle concentration in the composite system controlled at 100 µg mL^−1^.

### 4.11 Fabrication of Microneedles

Taking C@MN as an example, HAMA hydrogel microneedles were fabricated using PDMS microneedle molds via a casting‐negative pressure‐assisted molding method. Briefly, the HAMA precursor solution containing LAP was dropped onto the surface of the PDMS mold and subjected to negative pressure (−0.1 MPa) at room temperature to promote complete filling of the needle cavities and removal of trapped air bubbles. Excess solution on the mold surface was then removed, followed by mild heating at 30°C–35°C to allow moderate concentration/thickening of the precursor solution. To ensure sufficient filling of the needle tips, the drop‐casting and negative‐pressure steps could be repeated. After molding, ultraviolet irradiation (405 nm, 400 mW·cm^−2^) was applied to induce crosslinking and obtain stable HAMA microneedle structures. Subsequently, a 20% (w/v) PVA solution was dropped onto the back side of the mold as the backing layer. After drying at 30°C–35°C until the backing layer was formed, the microneedle patch was peeled off from the mold to obtain an intact HAMA hydrogel microneedle patch. Alternatively, PVA could be directly added after HAMA microneedle formation and drying, followed by photocuring (Figure 3A). Approximately 300 µL of 5% (w/v) HAMA hydrogel precursor solution containing 100 µg mL^−^^1^ Cu‐CeO_2_@ABs‐FTP was added to each microneedle mold, resulting in a Cu‐CeO_2_@ABs‐FTP loading of approximately 30 µg per microneedle patch.

### 4.12 In Vitro Antibacterial Performance Evaluation

Methicillin‐resistant *Staphylococcus aureus* (MRSA) and *Escherichia coli* (*E. coli*) were selected as representative Gram‐positive and Gram‐negative bacterial models, respectively. Samples from different treatment groups (Control, A@MN, C@MN, CA@MN, and CAF@MN) were co‐incubated with bacterial suspensions, with the initial bacterial concentration adjusted to 1.0 × 10^6^ CFU mL^−^^1^. Samples were placed in 24‐well plates, with 1 mL bacterial suspension added to each well, and incubated at 37°C. After incubation, the bacterial suspensions were serially diluted and uniformly spread onto agar plates, followed by further incubation at 37°C to observe colony formation, thereby qualitatively evaluating the inhibitory effects of different treatments on bacterial growth. Meanwhile, the absorbance of bacterial suspensions at 600 nm was measured, and the relative bacterial survival rate was calculated using the formula D_sample/D_control × 100%, where D_sample and D_control represent the absorbance values of the sample and control groups, respectively. In addition, to further analyze bacterial morphological changes after material treatment, bacterial samples from different groups were fixed, dehydrated through a graded series, and dried, followed by observation using SEM to evaluate the effects of the materials on bacterial cell structures. By combining colony formation results and SEM morphological observations, the in vitro antibacterial performance of each treatment group was comprehensively assessed.

### 4.13 Cell Culture

Primary human dermal fibroblasts (ATCC, Manassas, USA) and human skin fibroblasts (HSFs; IMMOCELL, Xiamen, Fujian, China) were cultured in Dulbecco's modified Eagle's medium (DMEM) supplemented with 10% fetal bovine serum and 1% penicillin‐streptomycin, with the addition of 10 ng mL^−1^ basic fibroblast growth factor (basic fibroblast growth factor, bFGF; Cat. No.: C044; Novoprotein, Shanghai, China) for routine culture. Cells were maintained at 37°C in a humidified atmosphere containing 5% CO_2_. To induce fibroblast‐to‐myofibroblast differentiation, cells were further cultured for 3 days in DMEM containing 10 ng mL^−1^ transforming growth factor‐β1 (transforming growth factor‐β1, TGF‐β1; MCE, USA).

### 4.14 Immunofluorescence Staining and Image Analysis

Cells were fixed with 4% paraformaldehyde (Beyotime, Shanghai, China) at room temperature for 10 min, followed by permeabilization with 0.1% Triton X‐100 (Beyotime, Shanghai, China) for 10 min. To detect α‐smooth muscle actin (α‐smooth muscle actin, α‐SMA) expression, cells were first blocked with 1% bovine serum albumin for 1 h at room temperature, then incubated with rabbit anti‐human α‐SMA primary antibody (Beyotime, Shanghai, China) overnight at 4°C, followed by incubation with Cy3‐conjugated goat anti‐rabbit IgG secondary antibody for 2 h at room temperature. Cells were subsequently stained for F‐actin and nuclei, with F‐actin labeled using Alexa Fluor 488‐conjugated phalloidin and nuclei counterstained with DAPI. All steps were interspersed with three washes in PBS. Finally, images were acquired using a confocal microscope, and the fluorescence intensity of α‐SMA was quantitatively analyzed using Fiji software.

### 4.15 Establishment of Full‐Thickness Skin Injury Models in Mice and Rabbits

All animal experiments in this study were approved by the Ethics Committee of Tongji University and conducted in accordance with relevant animal welfare regulations (Approval No.: TJAA09725501). Female BALB/c mice aged 6–8 weeks were purchased from Biotech‐Yuekang Life Technology (Shanghai) Co., Ltd. Prior to surgery, mice were anesthetized by inhalation of 3% isoflurane using an ABM small animal anesthesia system (Shanghai Yuyan Instruments Co., Ltd.). After adequate anesthesia, dorsal hair was removed using a depilatory cream, and the surgical area was disinfected three times with iodophor. A circular area with a diameter of approximately 10 mm was marked on the dorsal skin using a circular template, and the epidermal and dermal tissues were excised with surgical scissors and ophthalmic forceps to establish a full‐thickness skin defect model. Hydrogel microneedle patches were then applied to the wound surface and fixed using a medical fixation film. After surgery, mice were housed individually and allowed free movement after recovery from anesthesia (Figure S26). Wounds were photographed on days 0, 3, 7, and 14 post‐surgery.

Three‐month‐old New Zealand white rabbits were subjected to hair removal, anesthesia, and skin disinfection using the same procedures as those for mice, and all operations were performed under sterile conditions. Standardized circular wounds with a diameter of approximately 1 cm were created on the ventral side of the rabbit ear. After removal of the epidermal and dermal tissues, the perichondrium was further excised to establish a hypertrophic scar model, with wound sites carefully avoiding the central auricular artery and surrounding veins. The remaining procedures were performed with appropriate modifications following the mouse full‐thickness skin injury model. Rabbits were housed individually after surgery, and wound images were recorded on days 0, 5, 10, 20, and 30 postoperatively. In both the mouse and rabbit wound models, the microneedle patches were applied only once immediately after wound establishment, and no replacement, reapplication, or additional administration was performed during the subsequent observation periods.

### 4.16 Preparation of Engineered Jurkat T Cells and ABs

Jurkat T cells were co‐incubated with 20 µM Ac_4_ManNAz for 48 h, during which N_3_ groups were introduced into glycoproteins and glycolipids on the cell membrane surface through the sialic acid metabolic pathway. After incubation, the cells were washed twice with DPBS and collected as Jurkat T‐N_3_ cells. Subsequently, the cells were cultured in serum‐free medium containing 1.0 µM staurosporine for 3 h to induce apoptosis. The culture supernatant was collected and centrifuged at 50 × g for 5 min twice to remove intact cells and large cellular debris, followed by centrifugation at 2000 × g for 10 min. The resulting pellet was resuspended in PBS to obtain ABs‐N_3_ (Figure 1H) [33]. To verify N_3_ modification, Jurkat T cells, Jurkat T‐N_3_ cells, ABs, and ABs‐N_3_ were incubated with DBCO‐FITC, and fluorescent labeling was achieved through copper‐free click chemistry between DBCO and N_3_. After thorough washing to remove unbound probes, FITC fluorescence was analyzed by flow cytometry. Compared with their respective unmodified controls, Jurkat T‐N_3_ and ABs‐N_3_ exhibited significantly enhanced FITC fluorescence signals, confirming the successful introduction of N_3_ groups onto the cell surface and their retention on the derived ABs (Figure 1K,L).

### 4.17 Cellular Uptake of Nanoparticles

RAW 264.7 macrophages were stimulated with lipopolysaccharide (LPS, 100 ng mL^−1^) for 24 h to induce M1 polarization. Cells were then seeded onto coverslips placed in 24‐well plates at a density of 5 × 10^4^ cells per well and cultured for 24 h, followed by co‐incubation with Cy5.5‐labeled nanoparticles (100 µg mL^−1^). After incubation, cells were washed three times with PBS to remove non‐internalized nanoparticles, fixed with 4% paraformaldehyde for 10 min, and washed again with PBS. Cell nuclei and cell membranes were stained with Hoechst 33342 and DiO, respectively, and incubated in the dark for 10–20 min. Finally, intracellular nanoparticle uptake was observed using a confocal laser scanning microscope.

### 4.18 Biofilm Disruption Assay

The disruption effects of different MN patches on bacterial biofilms were evaluated using crystal violet staining. MRSA and E. coli suspensions were adjusted to approximately 1 × 10^6^ CFU mL^−^^1^ and separately added into 24‐well plates, followed by static incubation at 37°C for 48 h to establish biofilms. After incubation, the supernatant was removed, and the wells were gently washed twice with sterile PBS to eliminate non‐adherent planktonic bacteria. Subsequently, CM from the Control, A@MN, C@MN, CA@MN, and CAF@MN groups was added, and the biofilms were further incubated for 24 h. After treatment, the culture medium was removed, and the wells were gently washed three times with PBS. The plates were air‐dried and stained with 0.1% crystal violet solution for 15 min. After removing the staining solution and washing with PBS, images were captured to record the biofilm staining in each group. Subsequently, 33% acetic acid was added to fully dissolve the crystal violet bound to the biofilms, and the absorbance was measured at 570 nm to quantify the residual biofilm biomass. Each experiment was independently repeated at least three times.

### 4.19 HUVEC Tube Formation Assay

Matrigel was thawed overnight at 4°C, and 100 µL of Matrigel was added to each well of a pre‐chilled 48‐well plate, followed by incubation at 37°C for 30 min to allow gelation. HUVECs were digested, resuspended, and seeded onto the solidified Matrigel surface at a density of 4 × 10^4^ cells/well. Subsequently, conditioned media from the Control, A@MN, C@MN, CA@MN, and CAF@MN groups were added, and the cells were incubated at 37°C with 5% CO_2_ for 6 h. The formed vascular‐like networks were then observed and imaged using an inverted microscope. ImageJ software was used to quantitatively analyze the total branch length, number of nodes, and number of junctions. Each experiment was independently repeated three times.

### 4.20 PS Masking and Macrophage M2‐Like Polarization Assay

To evaluate the effect of PS on the surface of ABs membranes on macrophage M2‐like polarization, Annexin V was used to mask PS on ABs membranes. Cu‐CeO_2_@ABs released from CA@MN were incubated with Annexin V in Ca^2^^+^‐containing binding buffer according to the manufacturer's instructions. After incubation, unbound Annexin V was removed by washing, and the resulting PS‐blocked CA@MN treatment solution was obtained. RAW264.7 macrophages were treated with 100 ng mL^−^^1^ LPS for 24 h to induce M1‐like polarization. After washing, the cells were respectively treated with conditioned media from the C@MN, CJ@MN, CA@MN, and PS‐blocked CA@MN groups and further cultured for 48 h. After treatment, the cells were collected, co‐stained with F4/80 and CD206 antibodies, and analyzed by flow cytometry to determine the proportion of F4/80^+^CD206^+^ cells, thereby evaluating the effects of different treatments on macrophage M2‐like polarization. Each experiment was independently repeated three times.

### 4.21 Western Blot Analysis

HDFs and myofibroblasts were treated with the corresponding conditioned media according to the protocols described above, with DMEM used as the Control group and CAF@MN‐conditioned medium used as the experimental group. Total proteins were extracted using RIPA lysis buffer supplemented with protease and phosphatase inhibitors, and protein concentrations were determined using the BCA assay. Equal amounts of protein samples were subjected to SDS‐PAGE electrophoresis and subsequently transferred onto PVDF membranes. After blocking with 5% BSA, the membranes were incubated overnight at 4°C with primary antibodies against PI3K, p‐PI3K, AKT, p‐AKT, SMAD7, SMAD2, p‐SMAD2, SMAD3, p‐SMAD3, and GAPDH. After washing, the membranes were incubated with corresponding HRP‐conjugated secondary antibodies, and protein signals were visualized using an ECL chemiluminescence detection reagent. The grayscale intensity of protein bands was quantified using ImageJ software. Three independent biological replicates were performed for each experiment.

### 4.22 Statistical Analysis

Statistical analysis and graphing were performed using GraphPad Prism 8.0 software. All quantitative data are presented as mean ± standard deviation. Relative expression data were normalized to the corresponding control group. Normality and homogeneity of variance were assessed prior to data analysis. For data satisfying normal distribution and homoscedasticity, one‐way analysis of variance (one‐way ANOVA) combined with appropriate post hoc multiple comparison tests was used to analyze differences among groups. For data that did not meet these assumptions, corresponding nonparametric tests were applied. A value of p < 0.05 was considered statistically significant. All experiments were performed at least three times (n ≥ 3).

## Author Contributions

**Hongyi Zhang**: writing – original draft, methodology, conceptualization, validation, investigation. **Jinwei Li**: formal analysis, validation, data curation. **Shan Hua**: validation, data curation. **Shengming Wu**: writing – review and editing, methodology, conceptualization, funding acquisition. **Chenlong He**: methodology, investigation. **Yuan Zhu**: validation. **Ming Yin**: investigation. **Han Zhou**: validation. **Huawei Liu**: investigation. **Chika Hasegawa**: conceptualization. **Jun Lyu**: investigation. **Keyue Xiao**: validation. **Hua Jiang**: writing – review and editing, funding acquisition, project administration. **Yilong Wang**: writing – review and editing, funding acquisition, supervision. **Yuxin Qian**: supervision, project administration.

## Funding

This work was supported by the Featured Department Foundation of Shanghai East Hospital (Grant Number 2024‐DFTS‐007), the Featured Clinical Discipline Project of Shanghai Pudong Fund (Grant Number PWYts2021‐07), the East Hospital affiliated to Tongji University introduced talent research startup Fund (Grant Number DFRC2019008), the National Natural Science Foundation of China (Grant Number 32271439), and the Postdoctoral Fellowship Program of CPSF (Grant Number GZC20251497).

## Ethics Statement

All the animal experiments were approved by the Ethics Committee of Tongji University (Approval No. TJAA09725501) and performed in accordance with institutional guidelines.

## Conflicts of Interest

The authors declare no conflicts of interest.

## Supporting information




**Supporting File**: advs77192‐sup‐0001‐SuppMat.docx.

## Data Availability

The raw single‐cell RNA sequencing data generated in this study have been deposited in the public database of the China National Center for Bioinformation (CNCB) under project accession number PRJCA069564, submission number subPRO101133, and GSA accession number CRA047050. The project is entitled ‘Dual‐targeting biomimetic nanozymes loaded microneedle patch promotes scarless wound healing through anti‐inflammatory and anti‐fibrotic effects’.

## References

[1] S. Joshi , M. Maan , P. Barman , et al., “Advances in Biomaterials for Wound Care Management: Insights From Recent Developments,” Advances in Colloid and Interface Science 343 (2025): 103563, 10.1016/j.cis.2025.103563. 40479779

[2] P. Martin , C. Pardo‐Pastor , R. G. Jenkins , and J. Rosenblatt , “Imperfect Wound Healing Sets the Stage for Chronic Diseases,” Science 386, no. 6726 (2024): adp2974, 10.1126/science.adp2974. PMC7617408 39636982

[3] H. Zhang , X. Lin , X. Cao , Y. Wang , J. Wang , and Y. Zhao , “Developing Natural Polymers for Skin Wound Healing,” Bioactive Materials 33 (2024): 355–376, 10.1016/j.bioactmat.2023.11.012. PMC10818118 38282639

[4] O. A. Peña and P. Martin , “Cellular and Molecular Mechanisms of Skin Wound Healing,” Nature Reviews Molecular Cell Biology 25, no. 8 (2024): 599–616, 10.1038/s41580-024-00715-1. 38528155

[5] F. S. Younesi , A. E. Miller , T. H. Barker , F. M. V. Rossi , and B. Hinz , “Fibroblast and Myofibroblast Activation in Normal Tissue Repair and Fibrosis,” Nature Reviews Molecular Cell Biology 25, no. 8 (2024): 617–638, 10.1038/s41580-024-00716-0. 38589640

[6] D. S. Foster , M. Januszyk , K. E. Yost , et al., “Integrated Spatial Multiomics Reveals Fibroblast Fate During Tissue Repair,” Proceedings of the National Academy of Sciences 118, no. 41 (2021): 2110025118, 10.1073/pnas.2110025118. PMC8521719 34620713

[7] M. Sharifiaghdam , E. Shaabani , R. Faridi‐Majidi , S. C. De Smedt , K. Braeckmans , and J. C. Fraire , “Macrophages as a Therapeutic Target to Promote Diabetic Wound Healing,” Molecular Therapy 30, no. 9 (2022): 2891–2908, 10.1016/j.ymthe.2022.07.016. PMC9482022 35918892

[8] W. Jiang , X. Pang , P. Ha , et al., “Fibromodulin Selectively Accelerates Myofibroblast Apoptosis in Cutaneous Wounds by Enhancing Interleukin 1β Signaling,” Nature Communications 16, no. 1 (2025): 3499, 10.1038/s41467-025-58906-z. PMC11993684 40221432

[9] S. Mascharak , H. E. desJardins‐Park , M. F. Davitt , et al., “Preventing Engrailed‐1 Activation in Fibroblasts Yields Wound Regeneration Without Scarring,” Science 372, no. 6540 (2021): aba2374, 10.1126/science.aba2374. PMC9008875 33888614

[10] C. He , M. Yin , H. Zhou , et al., “Magnetic Nanoactuator‐Protein Fiber Coated Hydrogel Dressing for Well‐Balanced Skin Wound Healing and Tissue Regeneration,” ACS Nano 19, no. 1 (2025): 1713–1731, 10.1021/acsnano.4c15647. 39749690

[11] M. G. Jeschke , F. M. Wood , E. Middelkoop , et al., “Scars,” Nature Reviews Disease Primers 9, no. 1 (2023): 64, 10.1038/s41572-023-00474-x. 37973792

[12] E. J. Hamson , F. M. Keane , S. Tholen , O. Schilling , and M. D. Gorrell , “Understanding Fibroblast Activation Protein (FAP): Substrates, Activities, Expression and Targeting for Cancer Therapy,” PROTEOMICS—Clinical Applications 8, no. 5‐6 (2014): 454–463, 10.1002/prca.201300095. 24470260

[13] R. Li , Y. Zhao , T. Liu , et al., “Nano‐Drug Delivery System Targeting FAP for the Combined Treatment of Oral Leukoplakia,” Drug Delivery and Translational Research 14, no. 1 (2024): 247–265, 10.1007/s13346-023-01397-6. 37526880

[14] S. Shahvali , N. Rahiman , M. R. Jaafari , and L. Arabi , “Targeting Fibroblast Activation Protein (FAP): Advances in CAR‐T Cell, Antibody, and Vaccine in Cancer Immunotherapy,” Drug Delivery and Translational Research 13, no. 7 (2023): 2041–2056, 10.1007/s13346-023-01308-9. 36840906

[15] L. Juillerat‐Jeanneret , P. Tafelmeyer , and D. Golshayan , “Regulation of Fibroblast Activation Protein‐α Expression: Focus on Intracellular Protein Interactions,” Journal of Medicinal Chemistry 64, no. 19 (2021): 14028–14045, 10.1021/acs.jmedchem.1c01010. 34523930

[16] Y. Sun , M. Ma , D. Cao , et al., “Inhibition of Fap Promotes Cardiac Repair by Stabilizing BNP,” Circulation Research 132, no. 5 (2023): 586–600, 10.1161/CIRCRESAHA.122.320781. 36756875

[17] S. U. Hettiarachchi , Y.‐H. Li , J. Roy , et al., “Targeted Inhibition of PI3 Kinase/mTOR Specifically in Fibrotic Lung Fibroblasts Suppresses Pulmonary Fibrosis in Experimental Models,” Science Translational Medicine 12, no. 567 (2020): aay3724, 10.1126/scitranslmed.aay3724. 33115948

[18] J. Lee , J. Byun , G. Shim , and Y.‐K. Oh , “Fibroblast Activation Protein Activated Antifibrotic Peptide Delivery Attenuates Fibrosis in Mouse Models of Liver Fibrosis,” Nature Communications 13, no. 1 (2022): 1516, 10.1038/s41467-022-29186-8. PMC8938482 35314685

[19] H. Meng , J. Su , Q. Shen , et al., “A Smart MMP‐9‐Responsive Hydrogel Releasing M2 Macrophage‐Derived Exosomes for Diabetic Wound Healing,” Advanced Healthcare Materials 14, no. 10 (2025): 2404966, 10.1002/adhm.202404966. 39955735

[20] X. Zhang , J. Yang , S. Ma , et al., “Functional Diversity of Apoptotic Vesicle Subpopulations From Bone Marrow Mesenchymal Stem Cells in Tissue Regeneration,” Journal of Extracellular Vesicles 13, no. 4 (2024): 12434, 10.1002/jev2.12434. PMC11025359 38634538

[21] T. Liu , B. Xiao , F. Xiang , et al., “Ultrasmall Copper‐Based Nanoparticles for Reactive Oxygen Species Scavenging and Alleviation of Inflammation Related Diseases,” Nature Communications 11, no. 1 (2020): 2788, 10.1038/s41467-020-16544-7. PMC7270130 32493916

[22] T. Waghule , G. Singhvi , S. K. Dubey , et al., “Microneedles: A Smart Approach and Increasing Potential for Transdermal Drug Delivery System,” Biomedicine & Pharmacotherapy 109 (2019): 1249–1258, 10.1016/j.biopha.2018.10.078. 30551375

[23] G. Yu , Y. Chen , N. Yang , et al., “Apoptotic Bodies Derived From Fibroblast‐Like Cells in Subcutaneous Connective Tissue Inhibit Ferroptosis in Ischaemic Flaps via the miR‐339‐5p/KEAP1/Nrf2 Axis,” Advanced Science 11, no. 24 (2024): 2307238, 10.1002/advs.202307238. PMC11200024 38639443

[24] W. Uminska , Z. Fekner , D. Kasinski , and M. Pokrywczynska , “Extracellular Vesicles From Mesenchymal Stem/Stromal Cells as Emerging Tools in Wound Healing: Mechanisms and Therapeutic Potential,” Stem Cell Research & Therapy 17, no. 1 (2025): 21, 10.1186/s13287-025-04813-5. PMC12798021 41351173

[25] Y. Yan , M. Li , L. Guo , et al., “Silk Fibroin Hydrogel With Recombinant Silk Fibroin/NT3 Protein Enhances Wound Healing by Promoting Type III Collagen Synthesis and Hair Follicle Regeneration in Skin Injury,” Materials Today Bio 33 (2025): 101957, 10.1016/j.mtbio.2025.101957. PMC12198047 40575657

[26] M. Rybka , Ł. Mazurek , J. Jurak , et al., “Keratin‐TMAO Dressing Accelerates Full‐Thickness Skin Wound Healing in Diabetic Rats via M2‐Macrophage Polarization and the Activation of PI3K/AKT/mTOR Signaling Pathway,” International Journal of Biological Macromolecules 310 (2025): 143313, 10.1016/j.ijbiomac.2025.143313. 40274140

[27] S. W. Jere , H. Abrahamse , and N. N. Houreld , “Interaction of the AKT and β‐Catenin Signalling Pathways and the Influence of Photobiomodulation on Cellular Signalling Proteins in Diabetic Wound Healing,” Journal of Biomedical Science 30, no. 1 (2023): 81, 10.1186/s12929-023-00974-8. PMC10515080 37735655

[28] S. W. Jere , N. N. Houreld , and H. Abrahamse , “Role of the PI3K/AKT (mTOR and GSK3β) Signalling Pathway and Photobiomodulation in Diabetic Wound Healing,” Cytokine & Growth Factor Reviews 50 (2019): 52–59, 10.1016/j.cytogfr.2019.03.001. 30890300

[29] D. G. You , G. T. Lim , S. Kwon , et al., “Metabolically Engineered Stem Cell–Derived Exosomes to Regulate Macrophage Heterogeneity in Rheumatoid Arthritis,” Science Advances 7, no. 23 (2021): abe0083, 10.1126/sciadv.abe0083. PMC8172131 34078596

[30] G. Dou , R. Tian , X. Liu , et al., “Chimeric Apoptotic Bodies Functionalized With Natural Membrane and Modular Delivery System for Inflammation Modulation,” Science Advances 6, no. 30 (2020): aba2987, 10.1126/sciadv.aba2987. PMC7439513 32832662

[31] K. Nagahama , Y. Kimura , and A. Takemoto , “Living Functional Hydrogels Generated by Bioorthogonal Cross‐Linking Reactions of Azide‐Modified Cells With Alkyne‐Modified Polymers,” Nature Communications 9, no. 1 (2018): 2195, 10.1038/s41467-018-04699-3. PMC5989231 29875358

[32] D. K. Jeppesen , Q. Zhang , J. L. Franklin , and R. J. Coffey , “Extracellular Vesicles and Nanoparticles: Emerging Complexities,” Trends in Cell Biology 33, no. 8 (2023): 667–681, 10.1016/j.tcb.2023.01.002. PMC10363204 36737375

[33] J. Chen , Z. Wang , S. Liu , et al., “Lymphocyte‐Derived Engineered Apoptotic Bodies With Inflammation Regulation and Cartilage Affinity for Osteoarthritis Therapy,” ACS Nano 18, no. 43 (2024): 30084–30098, 10.1021/acsnano.4c11622. 39403980

[34] P. Patil , K. A. Russo , J. T. McCune , et al., “Reactive Oxygen Species–Degradable Polythioketal Urethane Foam Dressings to Promote Porcine Skin Wound Repair,” Science Translational Medicine 14, no. 641 (2022): abm6586, 10.1126/scitranslmed.abm6586. PMC10165619 35442705

[35] P. Jiang , L. Zhang , X. Liu , et al., “Tuning Oxidant and Antioxidant Activities of Ceria by Anchoring Copper Single‐Site for Antibacterial Application,” Nature Communications 15, no. 1 (2024): 1010, 10.1038/s41467-024-45255-6. PMC10837451 38307902

[36] M. Soh , D.‐W. Kang , H.‐G. Jeong , et al., “Ceria–Zirconia Nanoparticles as an Enhanced Multi‐Antioxidant for Sepsis Treatment,” Angewandte Chemie International Edition 56, no. 38 (2017): 11399–11403, 10.1002/anie.201704904. 28643857

[37] D. Xia , Y. Guo , R. Xu , and N. Li , “Emerging Strategies for Nitric Oxide Production and Their Topical Application as Nanodressings to Promote Diabetic Wound Healing,” Journal of Nanobiotechnology 23, no. 1 (2025): 53, 10.1186/s12951-025-03135-1. PMC11776289 39881346

[38] R. Schuster , F. Younesi , M. Ezzo , and B. Hinz , “The Role of Myofibroblasts in Physiological and Pathological Tissue Repair,” Cold Spring Harbor Perspectives in Biology 15, no. 1 (2023): a041231, 10.1101/cshperspect.a041231. PMC9808581 36123034

[39] K. M. McAndrews , T. Miyake , E. A. Ehsanipour , et al., “Dermal αSMA^+^ Myofibroblasts Orchestrate Skin Wound Repair via β1 Integrin and Independent of Type I Collagen Production,” The EMBO Journal 41, no. 7 (2022): 109470, 10.15252/embj.2021109470. PMC8982612 35212000

[40] J. Massagué and D. Sheppard , “TGF‐β Signaling in Health and Disease,” Cell 186, no. 19 (2023): 4007–4037, 10.1016/j.cell.2023.07.036. PMC10772989 37714133

[41] Z.‐M. Zhuang , Y. Wang , Z.‐X. Feng , et al., “Targeting Diverse Wounds and Scars: Recent Innovative Bio‐Design of Microneedle Patch for Comprehensive Management,” Small 20, no. 18 (2024): 2306565, 10.1002/smll.202306565. 38037685

[42] M. Sang , M. Cho , S. Lim , et al., “Fluorescent‐Based Biodegradable Microneedle Sensor Array for Tether‐Free Continuous Glucose Monitoring With Smartphone Application,” Science Advances 9, no. 22 (2023): adh1765, 10.1126/sciadv.adh1765. PMC10413647 37256939

[43] W. Zhu , J. Mei , X. Zhang , et al., “Photothermal Nanozyme‐Based Microneedle Patch Against Refractory Bacterial Biofilm Infection via Iron‐Actuated Janus Ion Therapy,” Advanced Materials 34, no. 51 (2022): 2207961, 10.1002/adma.202207961. 36239263

[44] F. Wang , W. He , B. Dai , X. Zhang , and Y. Wen , “Recent Advances in Asymmetric Wettability Dressings for Wound Exudate Management,” Research 8 (2025): 0591, 10.34133/research.0591. PMC11729271 39810852

[45] M. G. Bezold , A. R. Hanna , B. R. Dollinger , et al., “Hybrid Shear‐Thinning Hydrogel Integrating Hyaluronic Acid With ROS‐Responsive Nanoparticles,” Advanced Functional Materials 33, no. 31 (2023): 2213368, 10.1002/adfm.202213368. PMC10723243 38107427

[46] M. A. Saifi and S. Seal , “Nanoceria, the Versatile Nanoparticles: Promising Biomedical Applications,” Journal of Controlled Release 338 (2021): 164–189, 10.1016/j.jconrel.2021.08.033. 34425166

[47] M. L. Ermini and V. Voliani , “Antimicrobial Nano‐Agents: The Copper Age,” ACS Nano 15, no. 4 (2021): 6008–6029, 10.1021/acsnano.0c10756. PMC8155324 33792292

[48] K. M. Vannella and T. A. Wynn , “Mechanisms of Organ Injury and Repair by Macrophages,” Annual Review of Physiology 79, no. 1 (2017): 593–617, 10.1146/annurev-physiol-022516-034356. 27959618

[49] Y. G. Kim , Y. Lee , N. Lee , M. Soh , D. Kim , and T. Hyeon , “Ceria‐Based Therapeutic Antioxidants for Biomedical Applications,” Advanced Materials 36, no. 10 (2024): 2210819, 10.1002/adma.202210819. 36793245

[50] S. Chen , A. F. U. H. Saeed , Q. Liu , et al., “Macrophages in Immunoregulation and Therapeutics,” Signal Transduction and Targeted Therapy 8, no. 1 (2023): 207, 10.1038/s41392-023-01452-1. PMC10200802 37211559

[51] G.‐B. Im , Y. G. Kim , T. Y. Yoo , et al., “Ceria Nanoparticles as Copper Chaperones That Activate SOD1 for Synergistic Antioxidant Therapy to Treat Ischemic Vascular Diseases,” Advanced Materials 35, no. 16 (2023): 2208989, 10.1002/adma.202208989. 36706357

[52] Y. Nakayama , S. Kon , D. Kurotaki , J. Morimoto , Y. Matsui , and T. Uede , “Blockade of Interaction of α9 Integrin With its Ligands Hinders the Formation of Granulation in Cutaneous Wound Healing,” Laboratory Investigation 90, no. 6 (2010): 881–894, 10.1038/labinvest.2010.69. 20308983

[53] A. Nyström , D. Velati , V. R. Mittapalli , A. Fritsch , J. S. Kern , and L. Bruckner‐Tuderman , “Collagen VII Plays a Dual Role in Wound Healing,” Journal of Clinical Investigation 123, no. 8 (2013): 3498–3509, 10.1172/JCI68127. PMC3726167 23867500

[54] Y. Dong , G. Jin , C. Ji , et al., “Non‐Invasive Tracking of Hydrogel Degradation Using Upconversion Nanoparticles,” Acta Biomaterialia 55 (2017): 410–419, 10.1016/j.actbio.2017.04.016. 28428038

[55] O. Morris , M. A. Elsawy , M. Fairclough , et al., “In Vivo Characterisation of a Therapeutically Relevant Self‐Assembling 18 F‐Labelled β‐Sheet Forming Peptide and its Hydrogel Using Positron Emission Tomography,” Journal of Labelled Compounds and Radiopharmaceuticals 60, no. 10 (2017): 481–488, 10.1002/jlcr.3534. PMC5601235 28623878

[56] T. Zivari‐Ghader , M.‐R. Rashidi , and M. Mehrali , “Biological Macromolecule‐Based Hydrogels With Antibacterial and Antioxidant Activities for Wound Dressing: A Review,” International Journal of Biological Macromolecules 279 (2024): 134578, 10.1016/j.ijbiomac.2024.134578. 39122064

[57] J. Yang , S. Xiao , J. Deng , et al., “Oxygen Vacancy‐Engineered Cerium Oxide Mediated by Copper‐Platinum Exhibit Enhanced SOD/CAT‐Mimicking Activities to Regulate the Microenvironment for Osteoarthritis Therapy,” Journal of Nanobiotechnology 22, no. 1 (2024): 491, 10.1186/s12951-024-02678-z. PMC11330606 39155382

[58] Y. Pang , F. M. Amona , X. Chen , et al., “Phytochemical Nanozymes Reprogram Redox for Balanced Antimicrobial and Regenerative Therapy in Acute and Chronic Diabetic Wounds,” Redox Biology 85 (2025): 103718, 10.1016/j.redox.2025.103718. PMC12205665 40513180

[59] Y. Guo , S. Ding , C. Shang , et al., “Multifunctional PtCuTe Nanosheets With Strong ROS Scavenging and ROS‐Independent Antibacterial Properties Promote Diabetic Wound Healing,” Advanced Materials 36, no. 8 (2024): 2306292, 10.1002/adma.202306292. 37723937

[60] E. Shirakami , S. Yamakawa , and K. Hayashida , “Strategies to Prevent Hypertrophic Scar Formation: A Review of Therapeutic Interventions Based on Molecular Evidence,” Burns & Trauma 8 (2020): tkz003, 10.1093/burnst/tkz003. PMC7175766 32341924

[61] Y. Zhang , S. Wang , Y. Yang , et al., “Scarless Wound Healing Programmed by Core‐Shell Microneedles,” Nature Communications 14, no. 1 (2023): 3431, 10.1038/s41467-023-39129-6. PMC10257705 37301874

[62] K. Chen , Y. Liu , X. Liu , et al., “Hyaluronic Acid‐Modified and Verteporfin‐Loaded Polylactic Acid Nanogels Promote Scarless Wound Healing by Accelerating Wound Re‐Epithelialization and Controlling Scar Formation,” Journal of Nanobiotechnology 21, no. 1 (2023): 241, 10.1186/s12951-023-02014-x. PMC10369727 37496007

[63] Y. Liu , M. Zhang , C. Wang , et al., “Human Umbilical Cord Mesenchymal Stromal Cell‐Derived Extracellular Vesicles Induce Fetal Wound Healing Features Revealed by Single‐Cell RNA Sequencing,” ACS Nano 18, no. 21 (2024): 13696–13713, 10.1021/acsnano.4c01401. 38751164

[64] E. Becht , L. McInnes , J. Healy , et al., “Dimensionality Reduction for Visualizing Single‐Cell Data Using UMAP,” Nature Biotechnology 37, no. 1 (2018): 38–44, 10.1038/nbt.4314. 30531897

[65] B. A. Shook , R. R. Wasko , G. C. Rivera‐Gonzalez , et al., “Myofibroblast Proliferation and Heterogeneity are Supported by Macrophages During Skin Repair,” Science 362, no. 6417 (2018): aar2971, 10.1126/science.aar2971. PMC6684198 30467144

[66] T. M. Bauer , K. D. Mangum , S. D. Buckley , et al., “TNF‐α Represses Fibroblast to Myofibroblast Transition Through the Histone Methyltransferase Setdb2,” JCI Insight 10, no. 22 (2025): 190836, 10.1172/jci.insight.190836. PMC12643505 41277557

[67] S. K. Parapuram , X. Shi‐wen , C. Elliott , et al., “Loss of PTEN Expression by Dermal Fibroblasts Causes Skin Fibrosis,” Journal of Investigative Dermatology 131, no. 10 (2011): 1996–2003, 10.1038/jid.2011.156. 21654839

[68] S. Fu , S. Yi , Q. Ke , K. Liu , and H. Xu , “A Self‐Powered Hydrogel/Nanogenerator System Accelerates Wound Healing by Electricity‐Triggered on‐Demand Phosphatase and Tensin Homologue (PTEN) Inhibition,” ACS Nano 17, no. 20 (2023): 19652–19666, 10.1021/acsnano.3c02561. 37820299

[69] J. M. Elizarraras , Y. Liao , Z. Shi , Q. Zhu , A. R. Pico , and B. Zhang , “WebGestalt 2024: Faster Gene Set Analysis and New Support for Metabolomics and Multi‐Omics,” Nucleic Acids Research 52, no. W1 (2024): W415–W421, 10.1093/nar/gkae456. PMC11223849 38808672

[70] M. Kanehisa , M. Furumichi , Y. Sato , Y. Matsuura , and M. Ishiguro‐Watanabe , “KEGG: Biological Systems Database as a Model of the Real World,” Nucleic Acids Research 53, no. D1 (2025): D672–D677, 10.1093/nar/gkae909. PMC11701520 39417505

[71] D. Lv , Z. Xu , P. Cheng , et al., “S‐Nitrosylation‐Mediated Coupling of DJ‐1 With PTEN Induces PI3K/AKT/mTOR Pathway‐Dependent Keloid Formation,” Burns & Trauma 11 (2023): tkad024, 10.1093/burnst/tkad024. PMC10729783 38116467

[72] I. Correia‐Sá , A. Paiva , C. M. Carvalho , and M. A. Vieira‐Coelho , “Cutaneous Endocannabinoid System: Does it Have a Role on Skin Wound Healing Bearing Fibrosis?,” Pharmacological Research 159 (2020): 104862, 10.1016/j.phrs.2020.104862. 32454223

