# Supplementary material for: Dual‐Targeting Biomimetic Nanozymes Loaded Microneedle Patch Promotes Scarless Wound Healing Through Anti‐Inflammatory and Anti‐Fibrotic Effects
Source: Adv Sci (Weinh). 2026 Aug 17:e77192. Online ahead of print. doi: 10.1002/advs.77192 (PMC13479856; doi:10.1002/advs.77192)
Supplement: Supplementary file 1 [file ADVS-9999-e77192-s001.docx]

**Supporting Information**

**Dual-Targeting Biomimetic Nanozymes Loaded Microneedle Patch Promotes Scarless Wound Healing Through Anti-Inflammatory and Anti-Fibrotic Effects**

*Hongyi Zhang^1^*‡*, Jinwei Li^2^*‡*, Shan Hua^1^*‡*, Shengming Wu^3^*, Chenlong He^3^, Yuan Zhu^1^, Ming Yin^3^, Han Zhou^3^, Huawei Liu^3^, Chika Hasegawa^1^, Jun Lyu^4^, Keyue Xiao^3^, Hua Jiang^1^*, Yilong Wang^3^*, Yuxin Qian^1^**

1. Department of Plastic Surgery, Ear Reconstruction Institute, Shanghai East Hospital, School of Medicine, Tongji University, Shanghai, 200120, China.

2. Department of Pediatric Neurosurgery, Xinhua Hospital Affiliated to Shanghai Jiao Tong University School of Medicine, Shanghai, China.

3. Department of Plastic Surgery, Ear Reconstruction Institute, State Key Laboratory of Cardiology and Medical Innovation Center, Shanghai East Hospital, The Institute for Biomedical Engineering & Nano Science, School of Medicine, Tongji University, Shanghai, 200092, P. R. China.

4. Massachusetts General Hospital, Harvard University, Boston, 02114, USA.

‡ Contributed equally.

* Corresponding to:

Yuxin Qian; E-mail: heliosqyx@163.com

Yilong Wang; E-mail: yilongwang@tongji.edu.cn

Hua Jiang; E-mail: dosjh@126.com

Shengming Wu; E-mail: wushengmingqust@163.com

Keywords: Skin wound healing, Nanozymes, Inflammation, Anti-Fibrosis, Fibroblast activation protein

**Supplementary Figures**

**Figure S1.** TEM image of CeO_2_ nanoparticles showing a uniform, nearly spherical morphology with good dispersion. Scale bar = 100 nm.

**Figure S2.** SEM image of CeO_2_ nanoparticles showing a uniform spherical morphology. Scale bar = 100 nm.

**Figure S3.** Schematic illustration of the synthesis of Cu-CeO_2_.

**Figure S4.** Schematic illustration of the preparation of Cu-CeO_2_@ABs using a liposome extruder, in which Cu-CeO_2_ nanoparticle solution and ABs solution are repeatedly extruded to achieve ABs membrane coating.

**Figure S5.** Particle size distribution characterization of CeO_2_ (A), Cu-CeO_2_ (B), and Cu-CeO_2_@ABs (C), and zeta potential distribution characterization of CeO_2_ (D), Cu-CeO_2_ (E), and Cu-CeO_2_@ABs (F).

**Figure S6.** X-ray diffraction (XRD) pattern of CeO_2_.

**Figure S7.** X-ray Photoelectron Spectroscopy (XPS) Cu 2p spectra of Cu-CeO_2_.

**Figure S8.** Particle size distribution characterization of ABs (A) and ABs-N_3_ (B), and zeta potential distribution characterization of ABs (C) and ABs-N_3_ (D).

**Figure S9.** Comparison of the scavenging abilities of CeO_2_ and Cu-CeO_2_ toward O_2_•⁻ at different concentrations (n = 3). Data are means ± SD. *p < 0.05, **p < 0.01, ***p < 0.001, ns, p ≥ 0.05.

**Figure S10.** Comparison of the scavenging abilities of CeO_2_ and Cu-CeO_2_ toward •OH at different concentrations (n = 3). Data are means ± SD. *p < 0.05, **p < 0.01, ***p < 0.001, ns, p ≥ 0.05.

**Figure S11.** Comparison of the scavenging abilities of CeO_2_ and Cu-CeO_2_ toward H_2_O_2_ at different concentrations (n = 3). Data are means ± SD. *p < 0.05, **p < 0.01, ***p < 0.001, ns, p ≥ 0.05.

**Figure S12.** Comparison of oxygen concentration changes over time among different groups. Data are means ± SD. *p < 0.05, **p < 0.01, ***p < 0.001, ns, p ≥ 0.05.

**Figure S13.** Effects of different treatments on HUVECs migration, observed by scratch assay at 0 and 24 h (scale bar = 100 μm).

**Figure S14.** Quantitative analysis of HUVEC cell migration (n = 3). Data are means ± SD. *p < 0.05, **p < 0.01, ***p < 0.001, ns, p ≥ 0.05.

**Figure S15.** Evaluation of HUVEC tube formation based on different treatments. (A) Representative bright-field images of vascular-like networks formed by HUVECs on Matrigel after treatment with conditioned media from the Control, A@MN, C@MN, CA@MN, and CAF@MN groups. Scale bar = 100 μm. (B–D) Quantitative analysis of tube formation, including relative total branch length, relative number of nodes, and relative number of junctions (n = 3). Data are presented as mean ± standard deviation; ns, p ≥ 0.05; *p < 0.05; **p < 0.01; ***p < 0.001.

**Figure S16. Fabrication process of the hydrogel microneedle patch.** **A**: The HAMA hydrogel precursor solution was cast into the microneedle mold; **B**: Air bubbles in the precursor solution were removed by vacuum degassing under negative pressure (approximately −0.1 MPa) to ensure structural integrity of the microneedles; **C**: The mold was dried in a constant-temperature oven at 35 °C, after which PVA was added and the same procedure was repeated; **D**: The hydrogel microneedles were crosslinked by irradiation with 405 nm ultraviolet light; **E**: The solidified microneedles were demolded; **F**: The fabricated hydrogel microneedle patch. Note: FITC was incorporated into the microneedles for subsequent confocal Z-stack imaging, resulting in a green appearance.

**Figure S17. Schematic illustration of confocal Z-axis scanning of microneedles.** A laser beam scans the microneedles sequentially along the Z-axis from the substrate and needle base to the needle tips, acquiring optical cross-sectional images at different heights to enable three-dimensional reconstruction and structural analysis of the microneedles.

**Figure S18. Confocal Z-axis scanning images of the microneedle array.** Confocal microscopy was used to acquire sequential Z-stack images of the microneedle array. The images show intact and regularly arranged microneedles with continuous fluorescence distribution along the Z-axis, indicating good three-dimensional structural integrity of the microneedle array. Scale bar = 100 μm.

**Figure S19. Mechanical strength test of the microneedle patch.** **A**: Representative photograph of the microneedle patch during compression loading; **B**: Morphology of the microneedle patch after compression.

**Figure S20.** Quantitative analysis of needle length changes of CAF@MN at different time points (0, 10, 20, and 30 min) under 75% humidity at room temperature (n = 3). Data are means ± SD. *p < 0.05, **p < 0.01, ***p < 0.001, ns, p ≥ 0.05.

**Figure S21.** Inhibitory effects of CAF@MN on biofilm formation of MRSA and E. coli.

(A) Representative crystal violet staining images of MRSA and E. coli biofilms after treatment with Control, A@MN, C@MN, CA@MN, and CAF@MN groups. Scale bar = 5 mm. (B, C) Quantitative analysis of the effects of different treatments on biofilm formation of MRSA (B) and E. coli (C). Data are presented as mean ± standard deviation (n = 3). **p < 0.01; ***p < 0.001; ns indicates no statistically significant difference.

**Figure S22.** Immunofluorescence quantitative analysis of nanoparticle uptake by macrophages.

**Figure S23.** Flow cytometric analysis of nanoparticle uptake and macrophage M2-like polarization. (A) Flow cytometric analysis of FITC-labeled nanoparticle uptake by macrophages after treatment with C@MN, CH@MN, CJ@MN, CHA@MN, and CA@MN. (B) Flow cytometric analysis of CD206 expression in macrophages after different treatments.

**Figure S24.** Flow cytometry analysis of RAW264.7 cells and LPS-induced M1 macrophages.

**Figure S25.** Representative histograms of CD206-FITC fluorescence signals in macrophages after treatment with C@MN, CJ@MN, CA@MN, and PS-blocked CA@MN.

**Figure S26.** (A) A circular area with a diameter of approximately 1 cm was marked on the dorsal skin of mice using a stamp; (B) the skin tissue was excised along the marked area; (C) the established wound model, excised skin tissue, and pre-fabricated MN patch are shown, indicating good size matching between the MN patch and the wound; (D) the MN patch fully covered the wound area; (E) the MN patch and wound were secured using a medical fixation film to prevent displacement or detachment; (F) mice resumed free movement after surgery, with the MN patch remaining well fixed without detachment.

**Figure S27.** In vivo retention of CAF@MN at the wound site in mice. (A, B) Relative contents of Ce and Cu in wound tissues at days 1, 3, 5, 7, and 10 after CAF@MN application, determined by ICP-MS. (C) Quantitative analysis of fluorescence intensity at the wound site. (D) Representative in vivo fluorescence imaging images of mouse wound areas at the corresponding time points. Data are presented as mean ± SD. *p < 0.05; **p < 0.01; ***p < 0.001; ns, p ≥ 0.05.

**Figure S28.** Body weight and serum biochemical parameters (ALT, UREA, CK, GLU, and TG) of mice in different treatment groups remained within normal ranges at 1, 3, 7, and 14 days post-treatment, indicating good in vivo biosafety of the hydrogel dressing (n = 3). Data are means ± SD. *p < 0.05, **p < 0.01, ***p < 0.001, ns, p ≥ 0.05.

**Figure S29.** Comparison of T-SOD, GSH, CAT, and T-AOC levels in wound tissues of mice from different treatment groups on day 7 post-surgery, indicating enhanced antioxidant capacity with CAF@MN treatment (n = 3). Data are means ± SD. *p < 0.05, **p < 0.01, ***p < 0.001, ns, p ≥ 0.05.

**Figure S30.** H&E histological staining of the heart, liver, spleen, lung, kidney, and brain collected from mice at day 14 post-operation revealed no obvious pathological alterations, indicating good in vivo biocompatibility of the nanomaterials. Scale bar = 50 μm.

**Figure S31.** Representative SEM image of bacteria isolated from infected mouse wound tissues after LB culture, showing rod-shaped and coccoid morphologies consistent with typical features of E. coli and MRSA. Scale bar = 5 μm.

**Figure S32.** (A) Schematic illustration of the antibacterial mechanism of Cu-CeO_2_. (B) Representative images of bacterial colony formation on agar plates from mouse skin wound samples at day 3 after different treatments. Colonies from the Control group and the treated groups (A@MN, C@MN, CA@MN, and CAF@MN) were obtained from wound samples collected at day 3 post-operation and cultured on agar plates to evaluate the inhibitory effects of different treatments on wound-associated bacterial growth (MRSA and E. coli). Scale bar = 1 cm.

**Figure S33.** Heatmap of relative bacterial viability in vivo.

**Figure S34.** Establishment of the rabbit ear wound model and application of the microneedle patch. (A) A circular wound with a diameter of approximately 1 cm was created using a biopsy punch, with the depth reaching the perichondrium without damaging the cartilage; (B) removal of the skin, subcutaneous tissue, and perichondrium; (C) representative images of the established wound, excised skin tissue, and pre-fabricated MN patch; (D) the MN patch fully covered the wound area; (E) the MN patch was secured using a medical fixation film.

**Figure S35.** Lateral view images of rabbit ear skin wounds on postoperative day 30 for evaluation of scar tissue formation.

**Figure S36.** UMAP visualization of single-cell RNA sequencing data in the Control and CAF@MN groups (n = 3).

**Figure S37.** Bar plot showing the proportions of different cell types in two sample groups.

**Figure S38.** Volcano plots of differentially expressed genes in B cells, T cells, endothelial cells, smooth muscle cells, keratinocytes, and melanocytes.

**Figure S39.** GO-BP enrichment analysis of B cells, T cells, endothelial cells, smooth muscle cells, keratinocytes, and melanocytes.

**Figure S40.** Box plots illustrate and compare the expression level distributions of Smad7, Dcn, Socs3, Pten, Nfe2l2, and Fgf2 derived from single-cell RNA sequencing between the control and experimental groups.

**Figure S41.** GO-BP GSEA enrichment analysis of T cells and B cells.

**Figure S42.** Functional module association network of genes related to fibroblasts, myofibroblasts, and macrophages.

**Figure S43.** GSEA enrichment plot of the PI3K-AKT signaling pathway in myofibroblasts.

**Figure S44.** Effects of CAF@MN on protein expression associated with the PI3K/AKT and SMAD7/TGF-β/SMAD signaling pathways. (A) Western blot analysis of PI3K, p-PI3K, AKT, and p-AKT protein expression in HDFs treated with Control or CAF@MN. (B) Quantitative analysis of the relative expression levels of PI3K, p-PI3K, AKT, and p-AKT proteins. (C) Western blot analysis of SMAD7, SMAD2, p-SMAD2, SMAD3, and p-SMAD3 protein expression in myofibroblasts treated with Control or CAF@MN. (D) Quantitative analysis of the relative expression levels of the above proteins. GAPDH was used as the loading control. Data are presented as mean ± SD. *p < 0.05; **p < 0.01; ***p < 0.001; ns, p ≥ 0.05.

**Figure S45.** Expression levels of CD80 and CD206 across different single-cell populations.

**Figure S46.** Pseudotime distribution of lineages 1 and 2 in UMAP space.

**Figure S47.** Dynamic expression patterns of Pparg, Kl, Bmp7, Nfe2l2, Pten, Socs3, Dcn, and Fgf2 along pseudotime.

**Table S1.** Primer sequences used for RT-qPCR.

| Gene name | Forward primer (5’-3’) | Reverse primer (5’-3’) |
| --- | --- | --- |
| SMAD7 | GCCGAGGCGGATCTGAAGG | GATTCACAGCAACACAGCCTC |
| DCN | ACCTGAAGAACCTTCACGCA | CATTCCCTGGAAAGCCCCAT |
| SOCS3  PTEN  NFE2L2  FGF2  CD80  CD86  CD206 | ATGGTCACCCACAGCAAGTT  CTGCAGAAAGACTTGAAGGCG  AGGTTGCCCACATTCCCAAA  GCTGTACTGCAAAAACGGGG  ACCTGGGAAAAAACCCCCAGA  ATGGACCCCAGATGCACCAT  GTCAGAACAGACTGCGTGGA | GGTACTCGCTCTTGGAGCTG  TGCTTTGAATCCAAAAACCTTACT  AGTGACTGAAACGTAGCCGA  AGCCAGGTAACGGTTAGCAC  GACAACGATGACGACGACTGT  CGGCAGATATGCAGTCCCAT  AGGGATCGCCTGTTTTCCAG |
| CD163 | GATGCTTCCATCCAGTGCCT | CTCTGAATGACCCCCGAGGA |
| iNOS | GCCCCTGGAAGTTTCTCTTCA | CTGAGAACAGCACAAGGGGT |
| Arg1 | GTGAAGAACCCACGGTCTGT | AGAAAGGACACAGGTTGCCC |
| COL1A1 | TCTGCGACAACGGCAAGGTG | GACGCCGGTGGTTTCTTGGT |
| FN1 | GGTCCGGGACTCAATCCAAA | GACAGAGTTGCCCACGGTAA |
| FGFR1 | ACCAAACCGTATGCCCGTAG | CAGGTGGCATAACGGACCTT |
| COL1A2 | TGAACTTGTTGCTGAGGGCA | ACTGGGCCAATGTCCACAAA |
